# Peptide–MHC Class I Tetramers Can Fail To Detect Relevant Functional T Cell Clonotypes and Underestimate Antigen-Reactive T Cell Populations

**DOI:** 10.4049/jimmunol.1700242

**Published:** 2018-02-26

**Authors:** Cristina Rius, Meriem Attaf, Katie Tungatt, Valentina Bianchi, Mateusz Legut, Amandine Bovay, Marco Donia, Per thor Straten, Mark Peakman, Inge Marie Svane, Sascha Ott, Tom Connor, Barbara Szomolay, Garry Dolton, Andrew K. Sewell

**Affiliations:** * Division of Infection and Immunity, Cardiff University School of Medicine, University Hospital Wales, Cardiff CF14 4XN, United Kingdom;; † Department of Oncology and Ludwig Cancer Research, Lausanne University Hospital, Epalinges VD 1066, Switzerland;; ‡ Centre for Cancer Immune Therapy, Herlev University Hospital, DK-2730 Herlev, Denmark;; § Department of Immunobiology, Guy’s Hospital, King’s College London, London SE1 9RT, United Kingdom;; ¶ Department of Computer Science, University of Warwick, Coventry CV4 7AL, United Kingdom;; ‖ Systems Immunity Research Institute, Cardiff University School of Medicine, University Hospital Wales, Cardiff CF14 4XN, United Kingdom; and; # Cardiff University School of Biosciences, Cardiff CF10 3AX, United Kingdom

## Abstract

Peptide-MHC (pMHC) multimers, usually used as streptavidin-based tetramers, have transformed the study of Ag-specific T cells by allowing direct detection, phenotyping, and enumeration within polyclonal T cell populations. These reagents are now a standard part of the immunology toolkit and have been used in many thousands of published studies. Unfortunately, the TCR-affinity threshold required for staining with standard pMHC multimer protocols is higher than that required for efficient T cell activation. This discrepancy makes it possible for pMHC multimer staining to miss fully functional T cells, especially where low-affinity TCRs predominate, such as in MHC class II–restricted responses or those directed against self-antigens. Several recent, somewhat alarming, reports indicate that pMHC staining might fail to detect the majority of functional T cells and have prompted suggestions that T cell immunology has become biased toward the type of cells amenable to detection with multimeric pMHC. We use several viral- and tumor-specific pMHC reagents to compare populations of human T cells stained by standard pMHC protocols and optimized protocols that we have developed. Our results confirm that optimized protocols recover greater populations of T cells that include fully functional T cell clonotypes that cannot be stained by regular pMHC-staining protocols. These results highlight the importance of using optimized procedures that include the use of protein kinase inhibitor and Ab cross-linking during staining to maximize the recovery of Ag-specific T cells and serve to further highlight that many previous quantifications of T cell responses with pMHC reagents are likely to have considerably underestimated the size of the relevant populations.

## Introduction

Classically restricted T cells are mediators of adaptive immunity and recognize foreign peptides presented by MHC class I or II molecules displayed on the surface of APCs (1, 2). Typically, the foreign peptides that are recognized by T cells are derived from proteins expressed by pathogens; however, T cells also play a role in tumor surveillance by recognizing peptides derived from the dysregulated gene expression that occurs in cancer cells (3). The specificity of peptide–MHC (pMHC) recognition is conferred by the clonotypic αβ TCR, a heterodimeric cell surface receptor that is produced by somatic gene rearrangement of variable, diversity (of the β-chain), and joining segments at TCR loci, as well as nucleotide addition and/or deletion at recombination junctions (4). V(D)J gene rearrangement confers high variability to the CDR3 within a TCR, and, in unison with T cell cross-reactivity, allows TCR repertoires to recognize a vast number of potential foreign peptides (5). The development of fluorochrome-conjugated pMHC multimers enabled the visualization and phenotyping of Ag-specific T cells by flow cytometry and has transformed the study of T cell responses (6–9). The original, and still most commonly used, platform for pMHC multimers consists of four biotinylated pMHC molecules bound to fluorochrome-conjugated streptavidin (6). The resulting pMHC “tetramers” have been used in many studies and have become a standard feature of the T cell immunology toolkit (9). Typical staining protocols with pMHC tetramers fail to detect cognate T cells with weak TCR–pMHC affinity, because the affinity threshold for staining is higher than that required for T cell activation (10). Thus, regular pMHC tetramer staining fails to detect the T cells bearing lower-affinity TCRs that often predominate within antitumor and autoimmune T cell populations (7, 8, 11, 12). This problem is further compounded for MHC class II (MHCII)-restricted T cells, which are known to bear weaker-affinity TCRs than those raised against MHC class I (MHCI)-restricted peptides (13). In addition, unlike the MHCI–CD8 interaction (14), the MHCII–CD4 interaction does not aid binding of pMHC multimers (15), making staining with pMHCII multimers even more challenging than for pMHCI.

Much evidence suggests that T cells with low-affinity TCRs function relatively poorly (10, 16, 17), and the consensus view has become that T cells with higher-affinity TCRs stain better with pMHC multimers and exhibit greater sensitivity to cognate Ag. However, this assumption does not withstand close scrutiny, and many, more recent, studies demonstrate that pMHC multimers can fail to detect fully functional T cells (11, 12, 18, 19). Thus, staining with pMHC multimer is not a definitive surrogate marker of how sensitive a given T cell will be to cognate Ag or of how effective it might be in vivo. In accordance with this concept, Ploegh and colleagues (20) demonstrated that CD8 T cells with high- or low-affinity TCRs exhibited equivalent antitumor activity. Although the early results of Yee et al. (21) largely agreed with the view that the most effective T cells bear high-affinity TCRs and stain well using standard pMHC tetramer technology, it was noticeable that a minority of the highly functional cells in this study were poor at capturing pMHC tetramer from solution. Derby et al. (22) further showed that, although the functional sensitivity of T cells correlated with TCR-mediated signaling, it did not necessarily correlate with TCR affinity or pMHCI tetramer binding. These investigators concluded that caution should be exercised when directly relating TCR affinity and pMHCI tetramer staining to the functional sensitivity of T cells (22). Indeed, persistent human viral infections are known to drive out very large fully functional T cell populations (>5% of CD8 T cells) that cannot be detected by standard pMHCI tetramer staining (23). More recent studies by Evavold and colleagues (19, 24, 25) examining CD4 T cell populations in mice have been further illuminating. Staining with pMHCII tetramers was found to underestimate the H2-IA^b^–restricted CD4 T cell population specific for lymphocytic choriomeningitis virus glycoprotein_61–80_ by 4-fold and the H2-IA^b^–restricted population specific for myelin oligodendrocyte glycoprotein_35–55_ by 8-fold (19). Accordingly, these researchers concluded that the use of pMHC multimers has introduced a bias that underestimates the lower-affinity, but functional, components within diverse Ag-specific TCR repertoires (24). Further studies demonstrate that low-affinity T cells can represent major responders in primary CD4 T cell immune responses (25).

The reasons why fully functional T cells might fail to stain with cognate pMHC multimer are not completely understood but, in some cases, could be the result of recent activation in vivo. It is known that chronic stimulation of T cells during active disease can induce downregulation of the TCR (26), thereby hindering pMHC multimer staining of T cells bearing higher-affinity TCRs (12). In addition, the CD8 coreceptor, which binds to MHCI at a site distinct from the TCR-docking platform (14, 27), plays a key role in engagement of pMHCI multimers (28–30). Surface CD8 is also known to downregulate upon stimulation, detuning T cells and resulting in a loss of pMHC multimer binding (31). Consequently, failure to stain with standard pMHC multimer protocols could be the result of low surface density of a high-affinity TCR/coreceptor or due to expression of a TCR with lower affinity for the cognate Ag used in staining.

Various improvements to pMHC multimer staining methodologies aimed at reducing the TCR–pMHC affinity threshold for staining have been reported. These include the use of beneficial anti-coreceptor Abs (32–35), inhibiting TCR downregulation with protein kinase inhibitor (PKI) (36), the use of higher-order multimers with greater numbers of pMHC per molecule (11, 18), and stabilizing pMHC multimer binding by Ab cross-linking (12). These approaches can be applied with pMHCI and pMHCII reagents. Further MHC class–specific approaches have also been used, including enhancing the MHC interaction with the CD8 coreceptor (29) and use of improved peptide-flanking regions (37) to enhance staining with pMHCI or pMHCII multimers, respectively. Various combinations of the above-mentioned approaches have improved staining by up to 50-fold for some T cells and can result in considerable savings in terms of the concentration of pMHC multimer that is required for staining (7, 8).

We recently demonstrated that tumor-specific T cells in tumor-infiltrating lymphocyte (TIL) populations that failed to stain with pMHCI tetramers could be fully functional (12). Such T cells could be stained with pMHC multimers by a combination of applying a PKI during staining (36) and Ab cross-linking of pMHC reagent (12). We have also demonstrated that higher-order multimers, in the form of pMHC dextramers, could detect far more Ag-specific T cells than parallel staining with pMHC tetramers (11). Subsequently, Davis and colleagues (18) showed that another “next-generation” higher-order technology, pMHC dodecamers, which have 12 pMHC molecules per reagent, were able to detect 2–5-fold more Ag-specific human and murine CD4 and CD8 T cells compared with the equivalent tetramers. These low-affinity, tetramer-negative but dodecamer-positive, T cells were observed to exhibit comparable effector cytokine responses to the high-affinity cells that stained well with pMHC tetramer (18). Thus, the most recent staining protocols that have been developed enable detailed study of Ag-specific T cells that fail to stain with conventional pMHC multimers and confirm that regular staining with pMHC tetramers, as used in the bulk of studies, might miss the majority of the relevant T cell responses.

We use pMHC tetramer sorting in conjunction with high-throughput sequencing to analyze the TCRs used by populations of CD8 T cells specific for immunodominant epitopes from influenza, CMV, EBV, and insulin-like growth factor 2 mRNA-binding protein 2 (IMP2)-specific T cells in the blood of healthy donors. We additionally examined yellow fever virus–specific T cells in the blood of a vaccinated donor and melan A–specific T cells in patient TIL populations. We show that specific TCR clonotypes can be overlooked by standard pMHC tetramer staining and formally prove that these clonotypes can be fully functional. These studies further underscore the importance of using optimized protocols for pMHC multimer staining and suggest that many previous attempts to enumerate Ag-specific T cell populations by pMHC multimer staining will have underestimated the size of these populations.

## Materials and Methods

### Patients

Cryopreserved PBMCs from HLA A*0201 (HLA A2)-positive donors with type 1 diabetes were sourced and handled as previously described (38). TIL infusion product used to induce a complete lasting remission in stage IV malignant melanoma Patient MM909.24 and PBMCs taken postremission were procured as cryopreserved samples (39).

### T cell clones

The HLA A2–restricted EBV-specific CD8 T cell clone GD.GLC17 was cloned from a T cell line and recognizes the 9-mer BMLF-1–derived peptide GLCTLVAML (residues 280–288). CR0439.GLC was derived from the PBMCs of healthy Donor 0439 and also recognizes the GLCTLVAML peptide. The HLA A2–restricted CD8 T cell clone CR0439.NLS was derived from the same donor and recognizes a novel 10-mer peptide derived from IMP2 (residues 367–376; sequence NLSALGIFST).

The HLA A2–restricted melan A–specific CD8 T cell clones VB6G4.24, CR1, CR7, CR24, CR27, CR29, and CR31 recognize the wild-type 10-mer peptide EAAGIGILTV (residues 26–35) and its heteroclitic variant E**L**AGIGILTV (heteroclitic residue in bold) (40). MEL13 was grown from the PBMCs of a healthy donor and expressed an identical TCR to sister clone MEL5. The structure and biophysics of MEL5/MEL13 TCR in complex with HLA A2–EAAGIGILTV and HLA A2–E**L**AGIGILTV have been determined previously (40–42). All other melan A–specific CD8 T cell clones were derived from TILs of a stage IV malignant melanoma patient (MM909.24) who underwent therapy with TILs (39) and successfully cleared tumor. Tumor-reactive clone CR14 from MM909.24 did not stain with HLA A2–E**L**AGIGILTV tetramer and had unknown specificity. Clones were procured and routinely expanded as previously described (43).

### Lymphoblastoid cell lines and tumor cells

T2 cells and the Donor 0439 lymphoblastoid cell line (LCL) were cultured as suspension cells at 37°C and 5% CO_2_ in RPMI 1640 medium supplemented with 100 U/ml penicillin, 100 μg/ml streptomycin, 2 mM l-glutamine, and 10% FBS (R10; all from Life Technologies, Paisley, U.K.) and passaged weekly or as required.

Autologous melanoma cells of HLA A2^+^ Donor MM909.24 were cultured as an adherent monolayer at 37°C and 5% CO_2_ in R10 and detached from the tissue culture flask by gently washing with calcium and magnesium chloride–free Dulbecco’s PBS, followed by incubation with Dulbecco’s PBS and 2 mM EDTA at 37°C until cells detached.

### PBMCs and T cell lines

PBMCs were obtained from local healthy donors (heparinized) or buffy coats (EDTA treated) from the Welsh Blood Service, in accordance with appropriate ethical approval and informed consent. PBMCs were isolated using SepMate tubes (STEMCELL Technologies, Vancouver, BC, Canada), according to the manufacturer’s instructions, and Lymphoprep (Axis Shield, Oslo, Norway). To create T cell lines, PBMCs were cultured in priming medium (R10 with 10 mM HEPES buffer, 1× MEM nonessential amino acids, 1 mM sodium pyruvate [all from Life Technologies], and 20 IU/ml IL-2 (Proleukin; Prometheus, San Diego, CA) with 10^−5^ M LLWNGPMAV peptide from the nonstructural protein 4b of yellow fever virus (residues 214–222) and anti-human CD28 Ab (10 μg/ml) for 14 d.

### Flow cytometry and cell sorting

Dextramer-PE (Immudex, Copenhagen, Denmark) and premium-grade streptavidin-PE (catalog number S21388; Life Technologies) were used with monomeric pMHC as previously described (11). The same batches of streptavidin-PE and each individual pMHC were used throughout this study to avoid any possibility of batch-to-batch variation. Protease inhibitors (Merck, London, U.K.) and PBS (tetramers) or dextramer buffer were added to give a final pMHC multimer concentration of 0.1 mg/ml (with regard to the pMHC component), were stored in the dark at 4°C, and were used within 3 d of assembly. Generally, 0.4 μg of tetramer or dextramer was used per stain (4 μg/ml with regard to the pMHC component). Typically, 5 × 10^4^ cells of a T cell clone, 1–2 × 10^5^ TILs, and 2–3 × 10^6^ cells of a T cell line or PBMCs were stained per tube with dextramer or tetramer first on ice for 30 min. Cells were washed with PBS and then stained with LIVE/DEAD Fixable Violet Dead Cell Stain, ViViD (Life Technologies) for 5 min at room temperature, and then a mixture of Abs for 20 min on ice: anti-CD8–allophycocyanin (clone BW135/80; Miltenyi Biotec); anti-CD3–PerCP (clone BW264/56; Miltenyi Biotec); anti-CD19–Pacific Blue (clone HIB19; BioLegend), and anti-CD14–Pacific Blue (clone M5E2; BioLegend). Adapting the above, T cell clones and TILs were stained with ViViD, anti-CD8 Ab, and anti-CD3 Ab. PBMCs and T cell lines were gated sequentially: lymphocytes (forward and side scatter), single cells, viable (ViViD^neg^)/CD3^+^/CD14^neg^/CD19^neg^ cells, and then CD8^+^/pHLA multimer^+^ T cells were sorted for further analyses. TILs were gated similarly but did not include the dump channel for CD19^+^ and CD14^+^ cells. A FACSAria (Central Biotechnology Service, Cardiff University) was used for cell sorting, with desired cells directed into RNA extraction buffer (RNeasy Plus Micro Kit; QIAGEN, Heidelberg, Germany) or a single well of a 96-well U-bottom plate containing T cell media. Data were analyzed using FlowJo software (TreeStar).

### Standard and optimized pHLA multimers staining protocols

The standard protocol is described above (flow cytometry and sorting), with the optimized protocol featuring two additional steps: cells were treated with 50 nM dasatinib (PKI) at 37°C for 30 min and were not washed prior to staining with tetramer or dextramer (36), and post–pHLA staining and washing, 0.5 μg (10 μg/ml) of a mouse anti-PE primary unconjugated mAb (clone PE001; BioLegend) was used. The complete details have been described previously (12).

### TCR sequencing and analysis

RNA was extracted using an RNeasy Plus Micro Kit (QIAGEN), following the manufacturer’s instructions. The SMARTer RACE kit (Clontech, Paris, France) was used to generate full-length cDNA from TCR RNA, also following the manufacturer’s instructions. Two sets of primers (external and internal) were designed to perform a nested PCR of the CDR3 region of the TCR α and β genes. The PCR products were examined by agarose gel electrophoresis and purified by gel extraction before sample indexing. All samples were processed further to generate libraries for high-throughput Illumina sequencing. Libraries were processed with the NEBNext Ultra Library preparation kit (New England Biolabs, Cambridge, U.K.) and run on an Illumina MiSeq instrument using a MiSeq v2 reagent kit (Illumina, Cambridge, U.K.). TCR gene usage was determined using reference sequences from the ImMunoGeneTics database (http://www.imgt.org), and all TCR gene segments were designated according to ImMunoGeneTics nomenclature using MiXCR software (v1.8.1) (44). When cells were sorted from PBMCs for TCR sequencing, only sequences that were present in at least five reads were included in the analyses. Unless otherwise stated, we only included clonotypes present at >1% of the total reads when sequencing tetramer^+^ cells from cultured cell lines. Functional T cells sorted from the TIL infusion product and blood of patient MM909.24 were sequenced, and all TCRs used for analyses. Public TCRs were assigned using a curated database of TCR sequences with known Ag specificity (45). TCR sequences are available at the VDJdb database (45): https://github.com/antigenomics/vdjdb-db/issues/243.

### T cell functional assays

^51^Cr release cytotoxicity assays were performed as previously described (12). Briefly, cells were labeled with ^51^Cr (PerkinElmer, Waltham, MA) and coincubated with T cells at the desired T cell/target cell ratio. Supernatants were harvested after 4 h, the amount of chromium released was assessed by scintillation counting, and percentage lysis was calculated as previously described (12). Sensitivity to peptide was determined by activation assay and ELISA, as previously described (40). Briefly, T cell clones (30,000 cells per well and in duplicate) were incubated overnight with 60,000 T2 cells, in the presence or absence of the desired peptide, at different concentrations. Supernatants were harvested, and MIP-1β was quantified by ELISA, according to the manufacturer’s instructions (R&D Systems). PBMCs were prescreened for Ag responsiveness by IFN-γ ELISPOT, as previously described (46). Briefly, ELISPOT plates (MSIPS4510; Merck Millipore, Burlington, MA) were coated with capture Ab [Human IFN-γ ELISpot^BASIC^ (ALP); Mabtech, Nacka Strand, Sweden] for 4 h at 37°C and blocked with R10 for 1 h at room temperature, and 0.2 × 10^6^ PBMCs were used per well and incubated overnight (37°C and 5% CO_2_) in the presence or absence of peptide (10^−5^ M). Biotinylated detection Ab and alkaline phosphatase–conjugated streptavidin were used as per the manufacturer’s instructions (Mabtech), and plates were developed with an Alkaline Phosphatase Conjugate Substrate Kit (Bio-Rad, Hercules, CA). Spots were counted using an AID ELISpot reader (Autoimmun Diagnostika, Strassberg, Germany). T cells from TILs and PBMCs sorted based on function were incubated (5 h) with 30 μM of TNF processing inhibitor-0 (TAPI-0) and anti-TNF (47) and anti-CD107a Abs prior to flow cytometry sorting, as previously described (48). This allowed viable T cells to be used for TCR sequencing.

## Results

### Standard and optimized pMHC multimer staining recover similar virus-specific T cell populations

**FIGURE 1.**
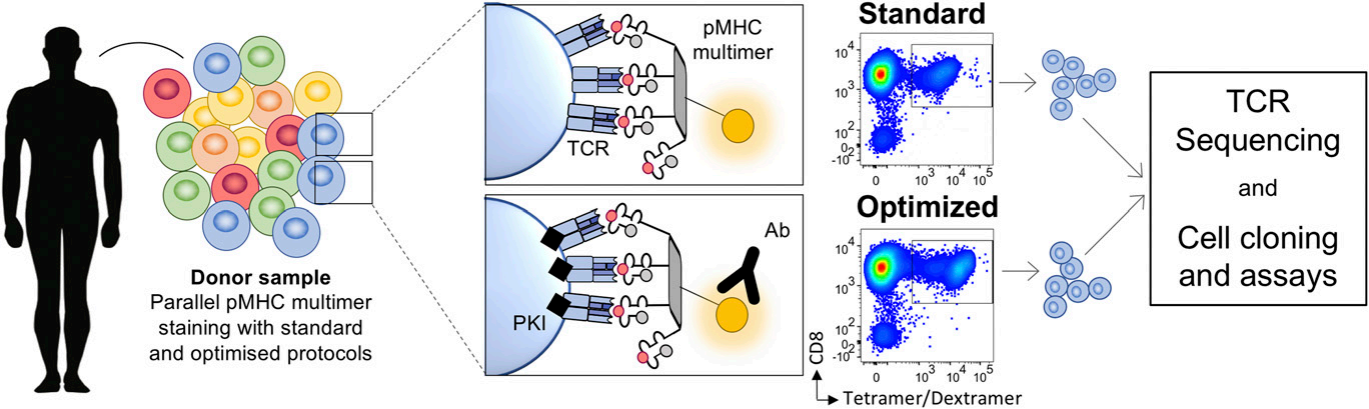
Study approach. Cell samples were stained in parallel using standard and optimized pMHC multimer staining protocols. The standard approaches used pMHC tetramer or dextramer, whereas the optimized protocol also included the PKI dasatinib and an anti-fluorochrome Ab. Multimer^+^ T cells were sorted by flow cytometry for TCR sequencing or cell cloning.

**FIGURE 2.**
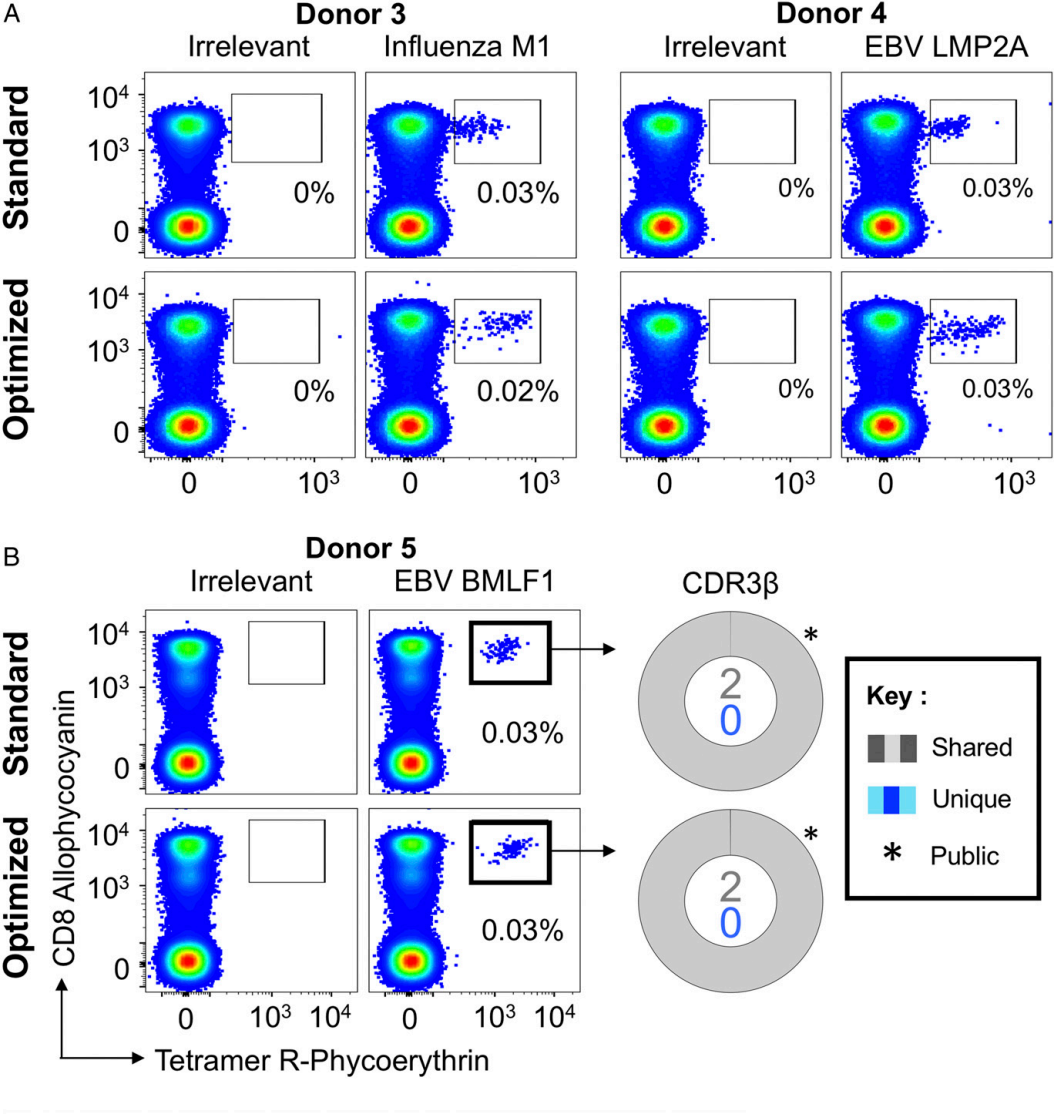
Ex vivo detection of Ag-specific CD8 T cells using standard pMHC multimer staining technology. (**A**) PBMCs from HLA A2^+^ healthy donors were stained with tetramers bearing the influenza M1_58–66_ peptide (GILGFVFTL; left panel) or the EBV LMP2A_426–434_ peptide (CLGGLLTMV; right panel), using tetramer alone (standard) or in combination with PKI and anti-fluorochrome Ab (optimized). Gates were set on single lymphocytes and live CD3^+^CD14^neg^CD19^neg^ cells. Irrelevant tetramer made with preproinsulin-derived peptide_15–24_ (ALWGPDPAAA) (65) or human telomerase reverse transcriptase (hTERT_540–548_, ILAKFLHWL) was used to set the gates. The percentage of CD8^+^Tet^+^ T cells is shown for each gate. (**B**) HLA A2^+^ PBMCs were sorted using EBV BMLF1_280–288_ (GLCTLVAML) standard tetramer staining and optimized staining protocols (left panel). CD8^+^Tet^+^ T cells were sorted, and TCR β-chain sequencing was performed to compare clonotype capture between protocols. TCR β-chains (right panel) are displayed as sort-shared (gray) or sort-unique (blue) sections of a pie, with each section for each sort corresponding to a different CDR3β. The number of shared (gray) and unique (blue) CDR3s for the respective sorts are shown in the center of each pie. TRBV usage is shown in Supplemental Fig. 2. A complete list of sorted β clonotypes can be found at https://github.com/antigenomics/vdjdb-db/issues/243.

Recent studies have suggested that pMHC multimer staining fails to detect fully functional T cells and, thereby, underestimates the size of Ag-specific T cell populations. It is becoming widely accepted that this problem is particularly pronounced when low-affinity TCRs predominate, such as in MHCII-restricted responses or those directed against self-antigens. Antiviral TCRs are known to generally bind with relatively high affinity (13, 49), and it has been assumed that standard pMHC tetramer–binding protocols are robust in detecting such cells. We made use of the pMHC tetramer staining strategies outlined in Fig. 1 to stain PBMCs from HLA A2^+^ donors who were known to make good responses to established immunodominant viral epitopes. Similar-sized populations of CD8 T cells were observed with standard and optimized staining for HLA A2–NLVPMVATV (CMV pp65) and HLA A2–CLGGLLTMV (EBV LMP2A) in Donors 1 and 2 (Supplemental Fig. 1A) and for HLA A2–GILGFVFTL (influenza matrix) and HLA A2–CLGGLLTMV in Donors 3 and 4 (Fig. 2A). We next examined the PBMCs of five donors using HLA A2–GLCTLVAML (EBV BMLF1). Donor 5 showed a similar pattern to that observed with the other viral epitopes (Fig. 2B), in that 0.03% of CD3^+^ cells were CD8^+^Tet^+^ with standard and optimized staining protocols. TCRβ sequencing of the CD8^+^Tet^+^ populations stained with each protocol generated just two sequences. The major sequence, TRBV29-1/TRBJ1-4 with CDR3 sequence CSVGTGGTNEKLFF, accounted for 99.96 and 99.92% of the reads with standard and optimized staining, respectively. This TRBV29-1/TRBJ1-4 chain is a well-described “public” TCR that has been isolated from other individuals (45). The other TCR β-chain, which made up <0.1% of total reads in each CD8^+^Tet^+^ gate, was TRB27/TRJB2-7 with CDR3 sequence CASTKTREKLYF. We conclude that these clonotypes stain well with standard pMHC tetramer staining protocols and that optimization using PKI and Ab cross-linking of pMHC tetramer offers little benefit for recovering these T cells from this donor. Importantly, although the CD8^+^Tet^+^ populations were of similar magnitude, with standard and optimized staining in all stains shown in Fig. 2 and Supplemental Fig. 1A, it was evident that the optimized protocol, which prevents “unproductive” engagement and triggering of TCRs (36) and lowers the off-rate of bound tetramer (12), stained cells with higher intensity and gave a better separation from the background CD8^+^Tet^neg^ population. While undertaking experiments to prove the usefulness of standard staining technologies for recovering T cells specific for immunodominant viral epitopes, we made the surprising discovery that, in most donors, the optimized protocol summarized in Fig. 1 detected large populations of CD8 T cells that bound to HLA A2–GLCTLVAML tetramer that were not observed when standard staining protocols were used. This potentially important finding warranted further investigation.

### Standard pMHC tetramer staining sometimes fails to detect virus-specific T cell populations

**FIGURE 3.**
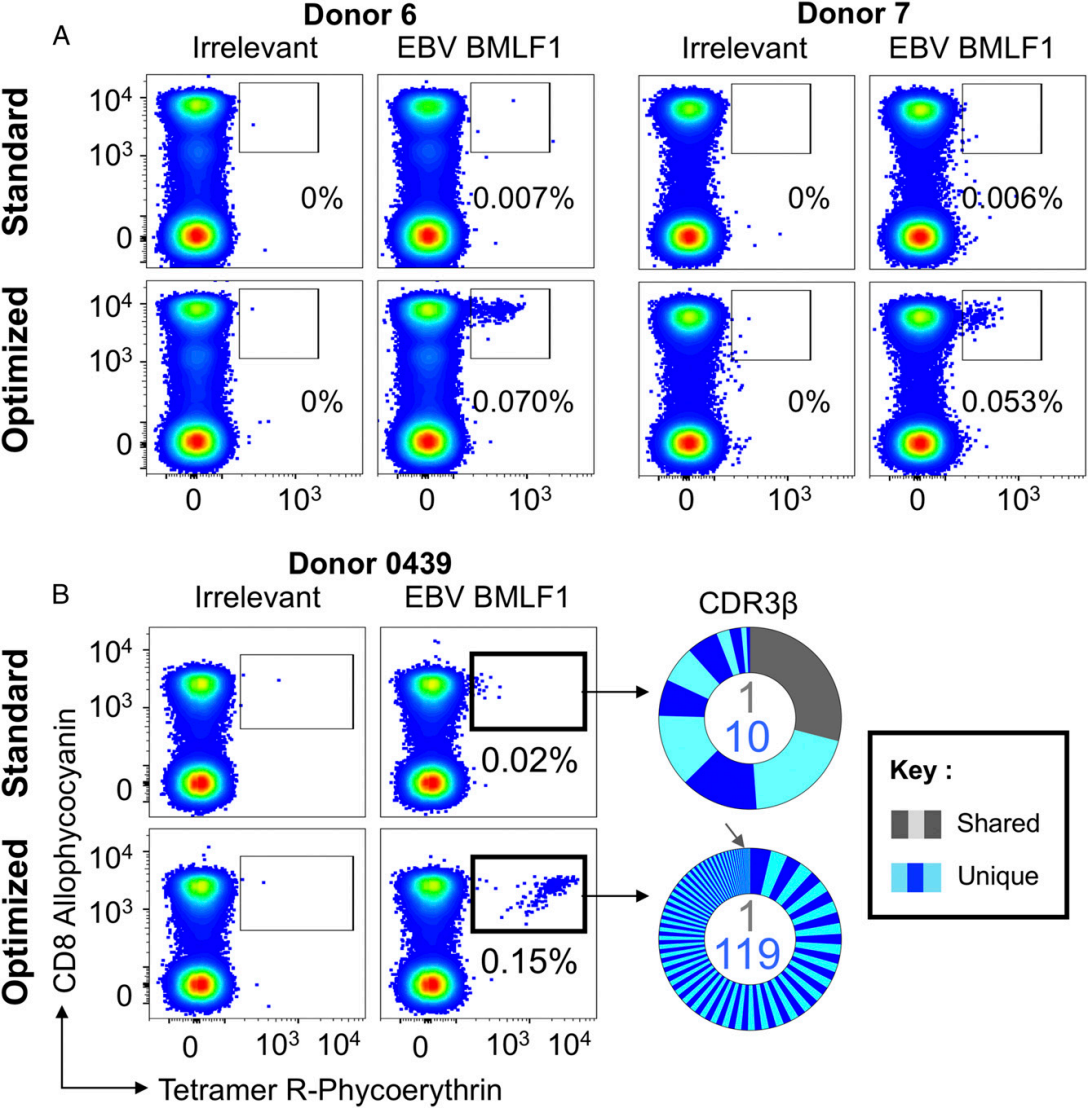
Optimized pMHC multimer staining revealed more EBV-specific clonotypes. PBMCs from HLA A2^+^ donors were stained ex vivo (**A**) and sorted (**B**) in parallel using HLA A2-GLCTLVAML (EBV, BMLF1_280–288_) tetramer alone (standard) or in combination with PKI and anti-fluorochrome Ab (optimized). Gates were set on lymphocytes and live CD3^+^CD14^neg^CD19^neg^ cells. Staining with irrelevant hTERT_540–548_ tetramer was used to set the gates. The percentage of CD8^+^Tet^+^ T cells is shown for each gate. β-chain TCRs of Donor 0439’s CD8^+^Tet^+^ sorted T cells (B, right panel) are displayed as sort-shared (gray) or sort-unique (blue) sections of a pie, with each section for each sort corresponding to a different CDR3β. The number of shared (gray) and unique (blue) CDR3s for the respective sorts are shown in the center of each pie. TRBV usage is shown in Supplemental Fig. 2. A complete list of sorted β clonotypes can be found at https://github.com/antigenomics/vdjdb-db/issues/243. The gray arrow in the optimized sort indicates the shared clonotype.

Six HLA A2^+^ donors who produced a good response to HLA A2–GLCTLVAML were stained with the cognate pMHC tetramer using the standard and optimized procedures outlined in Fig. 1. Staining produced similarly sized populations with each protocol in only one donor (Fig. 2, Donor 5). Surprisingly, this was not the situation in all of the other donors; the optimized protocol stained between 4 and 16 times more cells depending on the donor (Fig. 3, Supplemental Fig. 1B). Donor 0439 was of particular interest; multiple attempts to stain this donor’s cells with HLA A2–GLCTLVAML tetramer using regular protocols >15 y ago, when we were examining CD8 T cell populations specific for persistent DNA viruses (50), failed. This donor was not included in these studies, because they were scored as not responding to this epitope. The ELISPOT with GLCTLVAML peptide that we undertook to screen potential donors for this study clearly showed that PBMCs from Donor 0439 responded to this epitope (Supplemental Fig. 1C). The level of response observed by ELISPOT in Donor 0439’s PBMCs translated to ∼0.1% of CD3^+^ cells and did not equate to the lack of pMHC tetramer staining with this epitope that was observed previously in this individual. Subsequent staining with HLA A2–GLCTLVAML tetramer, using standard and optimized protocols, detected a substantial CD8^+^Tet^+^ population (0.15% of total CD3^+^ cells) when the optimized protocol was used. Staining with standard technology was very poor by comparison (Fig. 3B), explaining why we had previously assumed that this donor did not produce a response to GLCTLVAML peptide. TCRβ sequencing of the CD8^+^Tet^+^ populations showed that there were >100 clonotypes within the sorted CD8^+^Tet^+^ population from this donor. Ten of the eleven clonotypes sorted with the standard protocol were not observed in the population sorted with the optimized protocol. This may suggest that the GLCTLVAML-specific TCR repertoire is very large in this donor and, therefore, shows minimal overlap between replicate samples. However, given the very poor mean fluorescence intensity of staining of most cells within the sort gate with standard pMHC tetramer staining, this result is more likely to reflect capture of background clonotypes that are not GLCTLVAML-specific. Overall, this result indicates that standard pMHC tetramer staining with a viral CD8 T cell epitope can fail to detect functional T cell clonotypes and further serves to highlight what may have been missed in studies prior to the introduction of optimized tetramer staining techniques.

**FIGURE 4.**
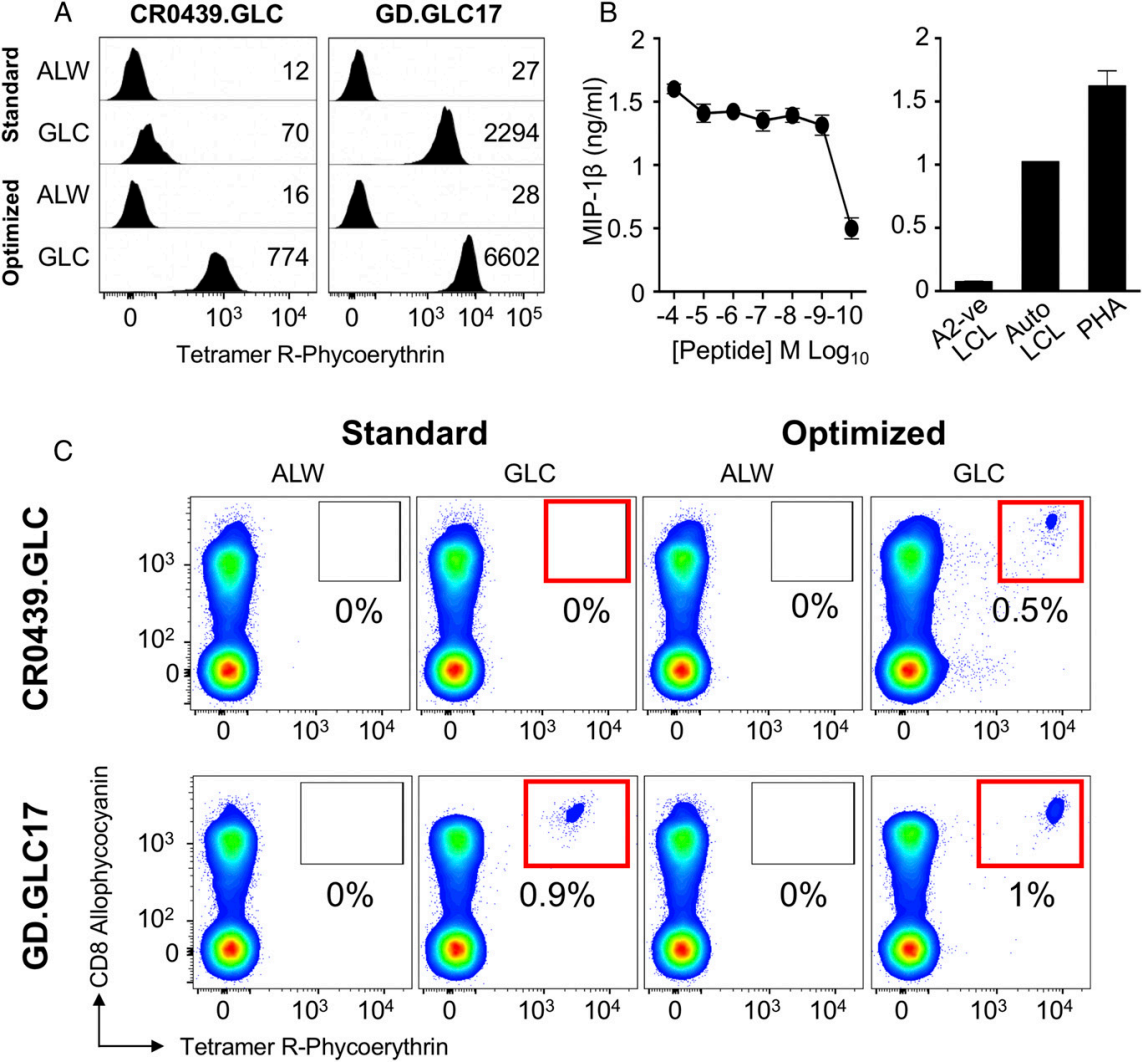
CD8 T cell clones grown from optimally EBV-tetramer stained and sorted PBMCs show effector function. (**A**) BMLF1-specific CR0439.GLC CD8^+^ clone (left panel) grown from the PBMCs of Donor 0439 and GD.GLC17 public CD8^+^ clone (right panel) staining with standard (tetramer-alone) and optimized (tetramer + PKI + anti-PE Ab) protocols for HLA A2–GLCTLVAML and irrelevant (ALWGPDPAAA from preproinsulin_15–24_) (65) tetramers. Mean fluorescence intensities are shown for each stain. (**B**) CR0439.GLC CD8^+^ clone exhibits functional activation when stimulated overnight with GLCTLVAML peptide (left panel) and robust recognition of EBV-infected cells (right panel), as measured by MIP-1β cytokine release. Uninfected cells and PHA were used as negative and positive controls, respectively. (**C**) Recovery of CR0439.GLC (upper panels) and GD.GLC17 (lower panels) clones spiked (aiming for 1% of CD3^+^ cell) into HLA A2^neg^ PBMCs by standard (red boxes, left panels) and optimized (red boxes, right panels) staining protocols with HLA A2-GLCTLVAML (BMLF1) and irrelevant HLA A2-ALWGPDPAAA.

To formally prove that functional T cell clonotypes were being missed during standard staining with EBV-specific pMHC tetramers, we set out to grow monoclonal populations from the CD8^+^Tet^+^ population that was sorted from Donor 0439 following optimized pMHC tetramer staining. In total, 755 single CD8^+^Tet^+^ cells were sorted into single wells for expansion across two experiments. Only 4 of 755 clones grew to a sufficient level for experimentation, but TCR sequencing showed that these had an identical TCR made from pairing a TRAV5/TRAJ15 chain (CDR3 CAEKGAGTALIF) and a TRBV29-1/TRBJ1-5 chain (CDR3 CSVAGTGDLNQPQHF). This clone presumably possesses a bias toward expansion in culture from the >100 clonotypes that were present in the CD8^+^Tet^+^ population. We have had similar results with antiviral T cells with other specificities. We do not know why only some T cell clones are adapted for in vitro culture, but it is possible that this might reflect a specific mutation in the clone or some aspect of the phenotype at the time of culturing. The clone generated was called CR0439.GLC, and it failed to stain with standard tetramer staining (Fig. 4A). Clone CR0439.GLC stained well with our optimized protocol, suggesting that this clone was representative of the T cell clones stained with the optimized, but not standard, protocol in Donor 0439 (Fig. 3B). A clone generated previously from another donor using standard pMHC tetramer staining, GD.GLC17 (expressing public TCR TRAV5/TRAJ31 chain, CDR3 CAEDNNARLMF and TRBV20-1/TRBJ1-3 chain, CDR3 CSARVGVGNTIYF), stained in parallel with both standard and optimized staining protocols.

We next set out to prove the CR0439.GLC clone, isolated from Donor 0439 with our optimized tetramer staining protocol, was fully functional. Peptide-titration assays showed that clone CR0439.GLC responded to cognate peptide at respectable exogenously applied concentrations < 1 nM (Fig. 4B) and could recognize the autologous EBV-infected LCL. These results confirm that standard pMHC tetramer staining misses fully functional antiviral T cell clonotypes. To emphasize this important point, the two clones stained in Fig. 4A were spiked into HLA A2^neg^ PBMCs at ∼1% total cells prior to tetramer staining with the optimized and standard protocols outlined in Fig. 1. Clone GD.GLC17 was recovered by both protocols, although staining was brighter with the optimized protocol. In contrast, none of the CR0439.GLC fully functional T cell clones could be recovered with standard pMHC tetramer staining. Conveniently, the CD8^high^ nature of cultured T cell clones, as described previously (11), allowed visualization of the clone within the PBMCs, without the need for tetramer staining (Fig. 4C). The CR0439.GLC clone spiked into PBMCs stained well when PKI and cross-linking Ab were included (Fig. 4C, upper right panel). We conclude that standard tetramer staining protocols, as have been used over the last 20 y, fail to detect fully functional antiviral T cells in some donors. With HLA A2–GLCTLVAML tetramers, this appeared to be the case in five of six donors tested in this study. We felt that it was important to extend our findings to another virus.

### Optimized pMHC multimer staining reveals more yellow fever–specific clonotypes than standard staining protocols

**FIGURE 5.**
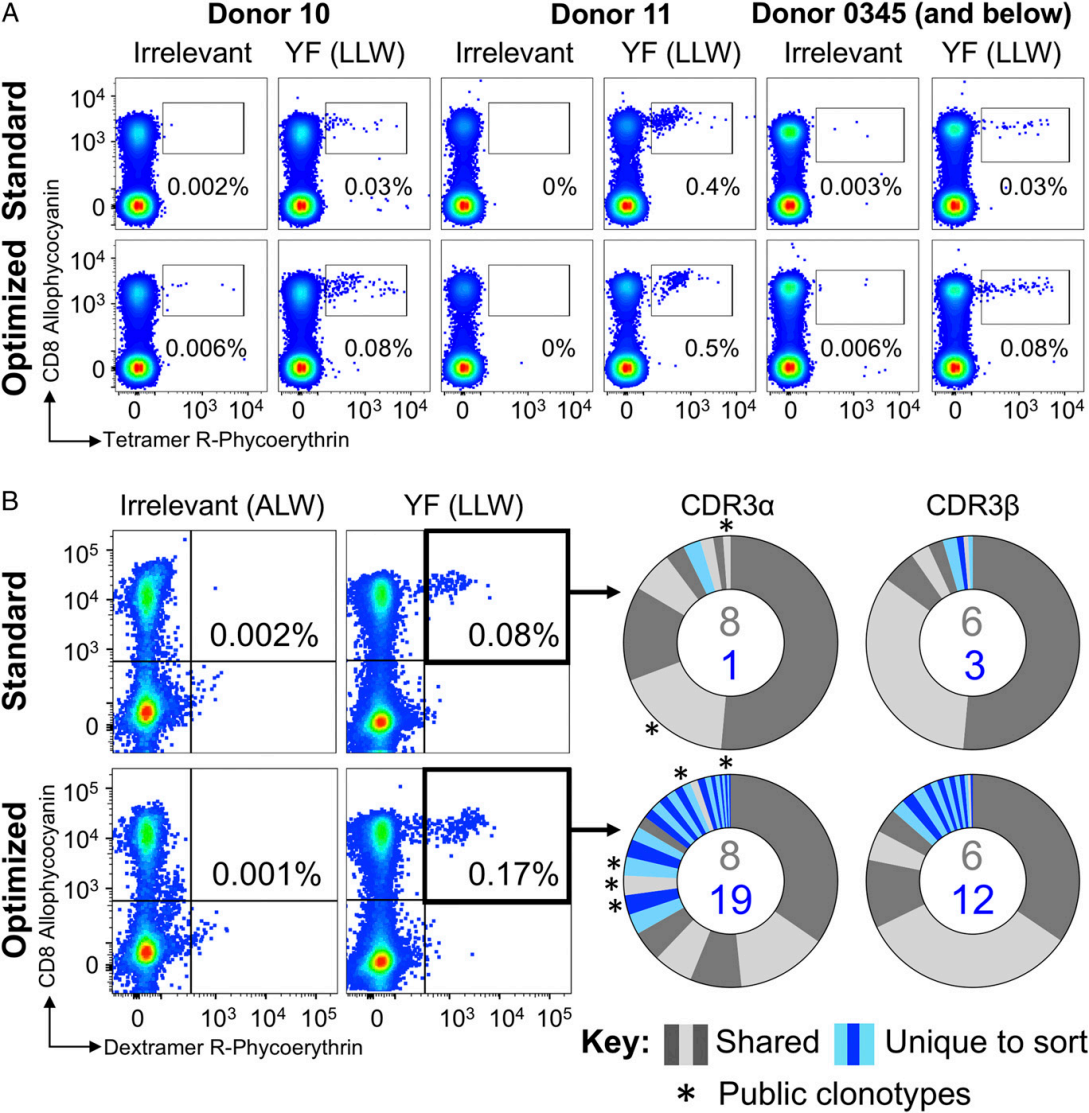
Optimized pMHC multimer staining revealed more yellow fever–specific clonotypes. (**A**) T cell lines from three HLA A2^+^ donors were stained with HLA A2 nonstructural protein 4b of yellow fever virus (residues 214–222) peptide (LLWNGPMAV) or irrelevant HLA A2-ALWGPDPAAA (preproinsulin_15–24_) (66) tetramers using standard (tetramer-alone) and optimized (tetramer + PKI + anti-PE Ab) protocols. (**B**) A T cell line from a yellow fever–vaccinated HLA A2^+^ donor (0345) was sorted by flow cytometry in parallel using HLA A2-LLWNGPMAV dextramer with standard or optimized staining as in (A). The percentage of dextramer^+^ cells of CD8^+^ T cells is shown for each gate. Irrelevant dextramer made with HLA A2–ALWGPDPAAA (65) was used to set the gates for sorting. TCR sequencing and CDR3 analysis of TCR α-chain and TCR β-chain are displayed as sort-shared (gray) or sort-unique (blue) sections of a pie, with each section for each sort corresponding to a different CDR3. The number of shared (gray) and unique (blue) CDR3s for the respective sorts are shown in the center of each pie. Public clonotypes are indicated by an asterisk.

We next stained a panel of T cell lines grown from the PBMCs of a vaccinated donor (Donor 0345) and nonvaccinated donors (Donors 10 and 11) with tetramers of the immunodominant HLA A2–restricted yellow fever virus epitope LLWNGPMAV (Fig. 5A). Consistent with previous observations with EBV BMLF1–specific CD8 T cells, more cells were detected with the addition of PKI and anti-fluorochrome Ab to the protocol. To examine the TCR clonotypes present in the HLA A2–LLWNGPMAV pMHC tetramer populations from yellow fever virus 17D–vaccinated Donor 0345, we made use of pMHC dextramers. We have previously shown that these higher-order multimers are better for staining T cells with low-affinity TCRs (11), allowing maximal recovery of LLWNGPMAV-specific cells for sequence analyses. We observed that standard staining of Donor 0345 cells with pMHC dextramer more than doubled the population of CD8 T cells that was stained compared with staining with tetramer [0.08% (Fig. 5B) compared with 0.03% (Fig. 5A) of CD3^+^ cells]. This proportion increased to 0.17% of cells when the PKI dasatinib was included with the anti-fluorochrome Ab in an optimized staining protocol (Fig. 5B). TRAV repertoire analyses of sorted cells by high-throughput sequencing revealed 9 CDR3s for the standard dextramer-stained cells (750 cells sorted) and 27 for the optimized staining (2066 cells sorted), with eight clonotypes shared between them (Fig. 5B, right panel). Importantly, the optimized staining protocol revealed 19 sort-unique CDR3s compared with one from the standard protocol sort (Fig. 5B). Similar analyses of TRBV use gave 9 and 18 CDR3s for standard and optimized staining, respectively, with six shared sequences. Thus, TCR β-chain sequencing also showed more CDR3s for the optimal protocol (*n* = 12) compared with the standard stained and sorted cells (*n* = 3) (Fig. 5). TRBV dominance was shared between TRBV20-1 and TRBV15 (Supplemental Fig. 2) and between TRBJ2-7 and TRBJ2-1, with a similar distribution seen for the standard and optimally sorted dextramer^+^ populations. Optimized HLA A2–LLWNGPMAV dextramer staining revealed public clonotypes (45, 51) that were not detected by standard dextramer staining (Fig. 5B, right panels, asterisks), further underscoring the importance of implementation of this methodology.

### Standard pMHC tetramer staining fails to detect tumor-specific T cell clones in TILs

**FIGURE 6.**
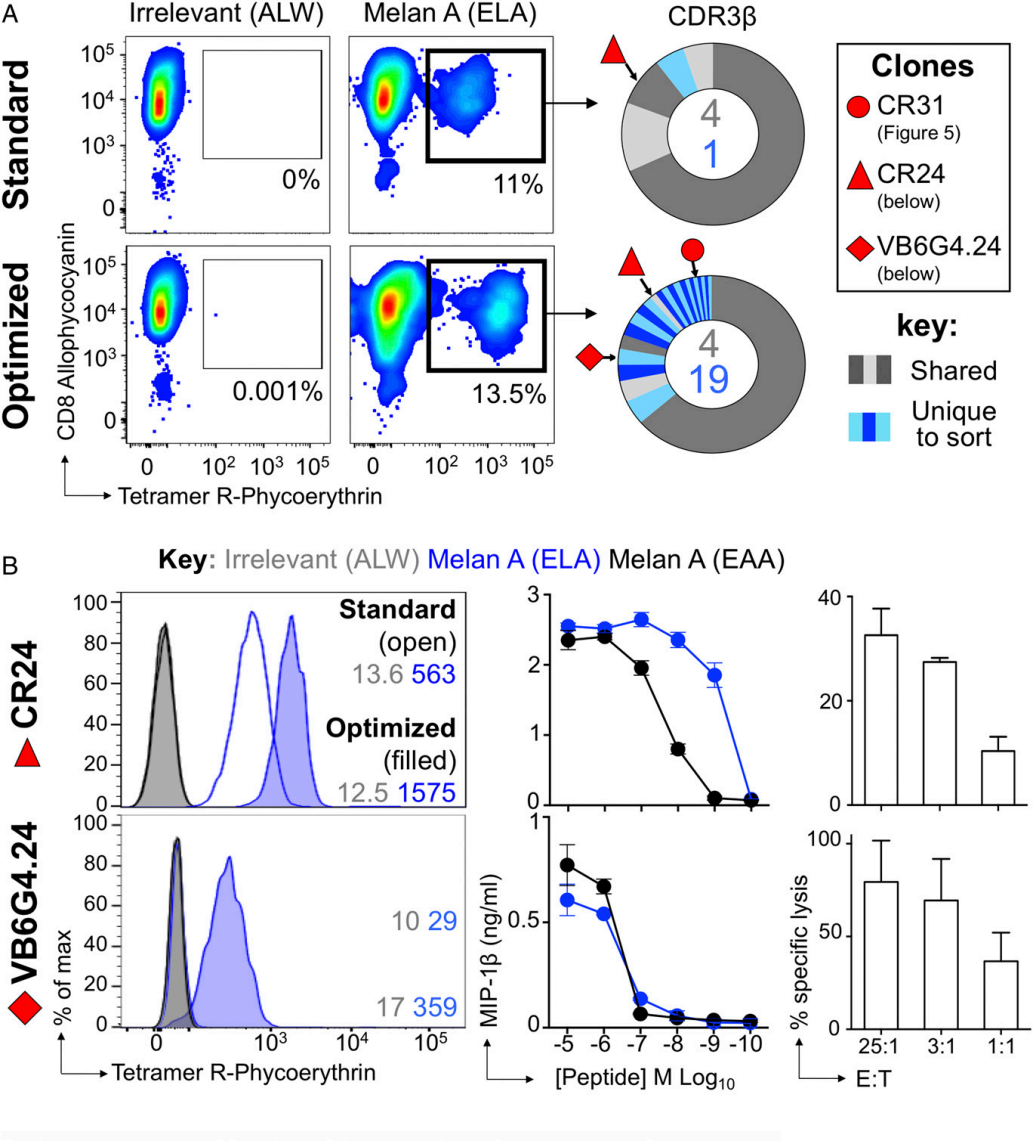
Clonotypic analysis of TILs using optimized pMHC staining reveals functional T cells missed by standard approaches. (**A**) Melan A–specific T cells were sorted from the TIL infusion product used to induce a complete remission in metastatic melanoma Patient MM909.24 using standard HLA A2–E**L**AGIGILTV (heteroclitic residue in bold) tetramer staining (tetramer-alone) and optimized (tetramer + PKI + anti-fluorochrome Ab) protocols. The percentage of tetramer^+^ cells is displayed. Irrelevant HLA A2–ALWGPDPAAA (66) was used to set the gates for sorting. TCR sequencing and CDR3 analysis of β-chains are displayed as sort-shared (gray) or sort-unique (blue) sections of a pie, with each section for each sort corresponding to a different CDR3. The number of shared (gray) and unique (blue) CDR3s for the respective sorts are shown in the center of each pie. (**B**) CDR3s of CD8^+^ clones grown from Patient MM909.24 are annotated. CD8^+^ clones CR24 and VB6G4.24 derived from Patient MM909.24 were stained with standard and optimized protocols using HLA A2–E**L**AGIGILTV (ELA) and irrelevant tetramers, with mean fluorescence intensities shown (left panels). Overnight activation against ELA and wild-type EAAGILGILTV (EAA) peptides (middle panels). Killing of autologous tumor by CR24 and VB6G4.24 at the E:T ratios shown (right panels).

We extended the study to a clinical sample by staining melanoma-derived TILs from patient MM909.24, who achieved complete lasting remission following adoptive transfer of “young TILs” at Herlev University Hospital (39). As previously described, standard and optimized pMHC tetramer staining approaches were used; consistent with our previous studies of this patient (12), more HLA A2–E**L**AGIGILTV tetramer^+^ T cells were detected with the optimized protocol that contained PKI and Ab cross-linking than without these additions (Fig. 6A). Again, this translated to a greater proportion of TRBV clonotypes being revealed, with 19 unique clones for the optimized protocol compared with only 1 for the standard protocol (Fig. 6A, right panel). The sorts shared four CDR3s, and usage for TRBV27 and TRBVJ2-3 dominated (>60% of total reads) (Supplemental Fig. 2).

### Clonotypes not revealed by standard tetramer staining are functionally relevant

The clonotyping of tetramer^+^ TILs revealed the TCR β-chain of CD8 T cell clone CR24 (TRBV6-5/TRBJ2-7; CDR3β, CASSYSFTEATYEQYF) in standard and optimally stained samples (red triangles, Fig. 6A). As expected, CR24 stained well with a standard tetramer protocol (Fig. 6B, upper left panel), hence its appearance in the standard-stained TILs. The TCR from the previously described (12) clone VB6G4.24 (TRBV24-1/TRBJ2-1; CDR3β, CATSDRGQGANWDEQF) appeared only in the optimally stained TILs (red rhombus, Fig. 6A) and required the optimized protocol for staining as a clone (Fig. 6B, lower left panel). Both of these clones recognize the wild-type peptide (EAAGIGILTV) and the commonly used heteroclitic version (E**L**AGIGILTV) (40) of the melan A/MART1 peptide (Fig. 6B, middle panels) and kill autologous tumor (Fig. 6B, right panels). Thus, the VB6G4.24 clone that cannot be stained by standard pMHC tetramer staining protocols and that is not recovered from TILs using such a protocol is functionally efficacious.

**FIGURE 7.**
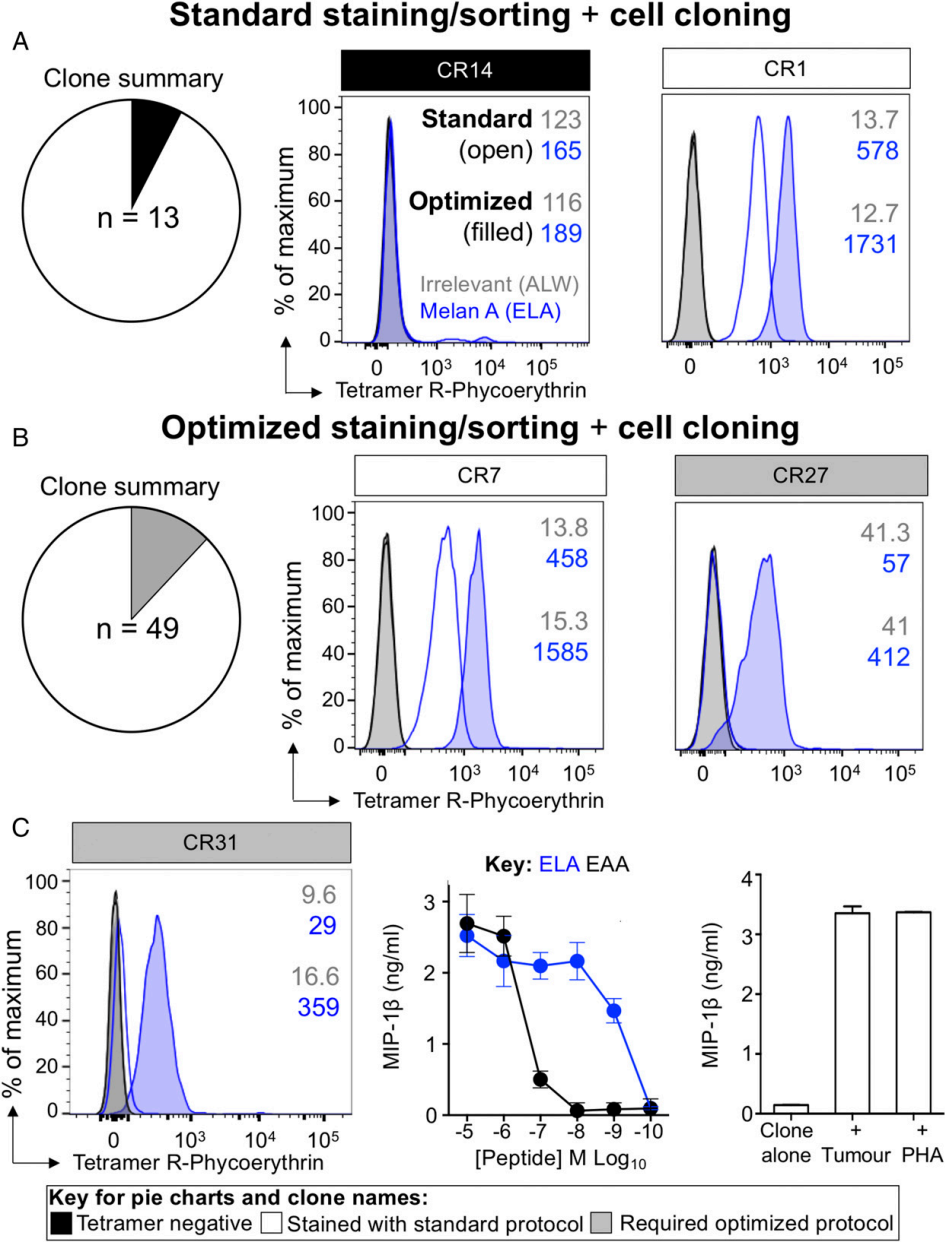
Clones grown from optimally stained and sorted TILs stain true for tetramer. Melan A–specific T cells were sorted from the TILs of metastatic melanoma Patient MM909.24, who achieved complete remission, using standard (tetramer-alone) (**A**) and optimized (**B**) protocols (tetramer + PKI + anti-fluorochrome Ab). Cells were cloned by limiting dilution and stained with HLA A2–E**L**AGIGILTV (heteroclitic residue in bold) and irrelevant HLA A2–ALWGPDPAAA tetramers using standard (A) and optimized (B) protocols. (A and B) Summary of the number of clones that grew and stained shown within the respective pie (left panel). Representative CD8^+^ clones encompassing all staining patterns seen (middle and right panels). (**C**) Patient MM909.24–derived CD8^+^ clone CR31, grown from optimally pMHCI–stained and sorted TILs, fails to stain using a standard protocol with HLA A2–E**L**AGIGILTV tetramer. HLA A2–ALWGPDPAAA irrelevant tetramer was used as control. Mean fluorescence intensities are displayed. CR31 responds well to ELA and wild-type EAAGIGILTV (EAA) peptides. CR31 exhibits robust recognition of autologous tumor cells. T cell alone and PHA as positive control are shown.

To further validate that the additional T cells revealed by optimized pMHC tetramer staining belong to clones that are functional, we performed further HLA A2–E**L**AGIGILTV tetramer-based sorts of MM909.24 TILs to grow clones, for tetramer staining and functional analysis, as outlined in Fig. 1. In total, 62 clones were grown, 13 from the standard stained TILs and 49 from the optimally stained TILs (Fig. 7A, 7B, left panels). Clones from the standard-stained TILs were tetramer negative (CR14; Fig. 7A, middle panel) or stained sufficiently with the standard protocol (CR1; Fig. 7A, right panel). Importantly, all of the clones from the optimized sort stained with tetramer using the optimized protocol, but only some of these clones stained with the standard protocol (Fig. 7B). Clone CR27 fell into the latter category; this clone stained only with an optimized protocol that included PKI and cross-linking of pMHC multimer with anti-fluorochrome Ab (Fig. 7B, right panel). Clonotypic analysis of clones that required the optimized protocol to stain, revealed that clone CR31 expressed a TCR β-chain that was only recovered from TILs with the optimized staining protocol (red circle, Fig. 6B; TRBV30/TRBJ1-5; CDR3β, CAWSSQGLGQPQHF). Although this clone failed to stain with standard pMHC tetramer staining (Fig. 7C, left panel), it was efficient at recognizing peptide (Fig. 7C, middle panel) and patient-autologous tumor cells (Fig. 7C, right panel).

**FIGURE 8.**
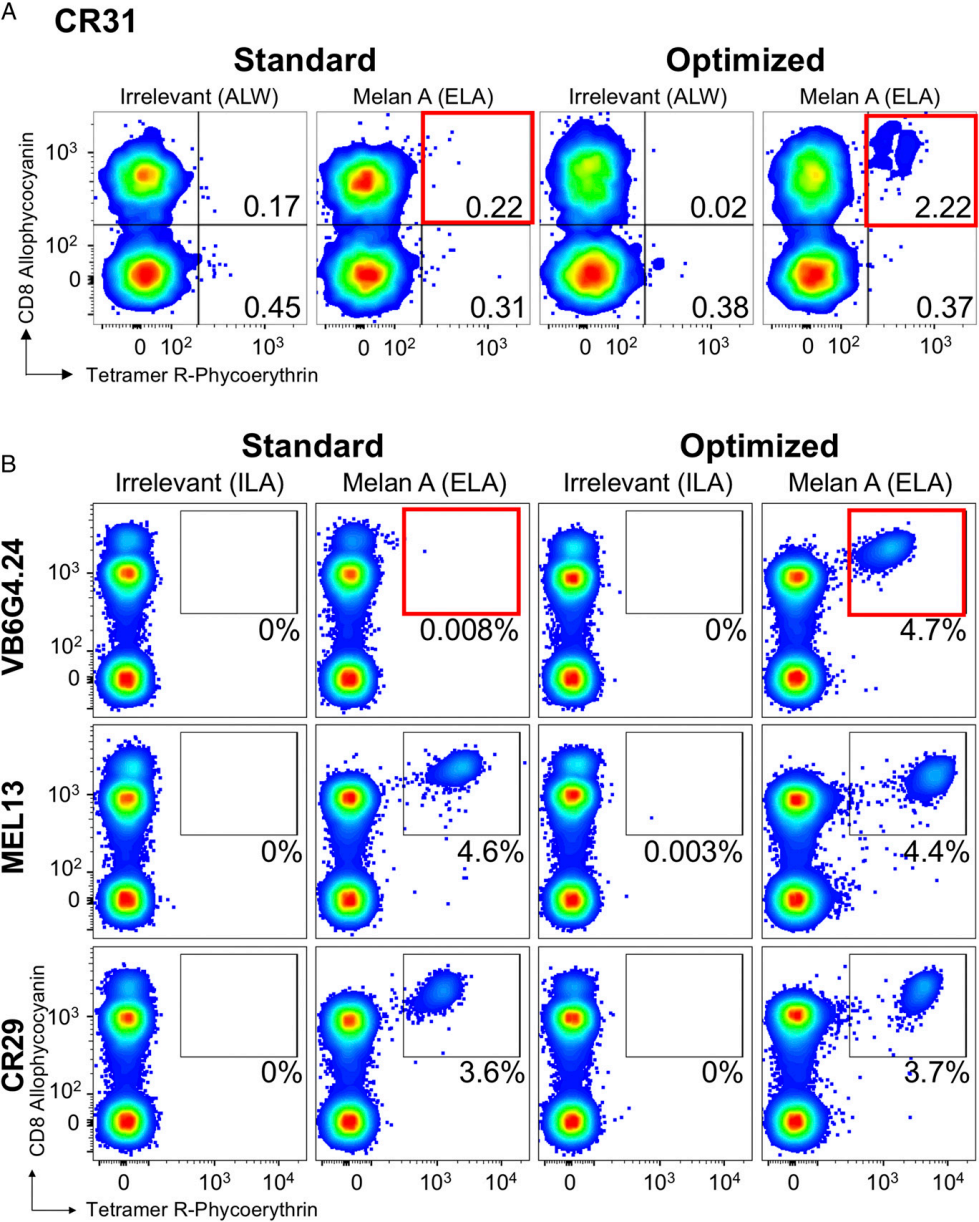
A standard tetramer protocol was not sufficient for detecting low-affinity melan A clones from PBMCs. (**A**) Recovery of CR31 spiked (aiming for 2% of CD3^+^ cell) into HLA A2^neg^ PBMCs by standard (tetramer-alone) and optimized (tetramer + PKI + anti-PE Ab) (red boxes) staining protocols with HLA A2–E**L**AGIGILTV (heteroclitic residue in bold) and irrelevant tetramers (ALWGPDPAAA from preproinsulin_15–24_). (**B**) Recovery of melan A–specific clones VB6G4.24 (top panels), MEL13 (middle panels), and CR29 (bottom panels) spiked into HLA A2^neg^ PBMCs (aiming for 5% of CD3^+^ cells) and stained using a standard or optimized staining protocol, as in (A), with HLA A2–E**L**AGIGILTV and irrelevant (ILAKFLHWL from hTERT_540–548_) (67) tetramers. Clone VB6G4.24 stains only with the optimized protocol (red box, top right panel). Gating: lymphocytes and then viable CD3^+^/CD14^neg^/CD19^neg^ cells. Plots are displayed as smoothed, with outliers and large dots. Percentages are shown for the respective gates. Spiked clones exhibit a high CD8 expression relative to those from PBMCs and can be visualized by CD8 staining alone.

We next spiked HLA A2^neg^ PBMCs with clone CR31 at ∼2% of CD3^+^ cells and stained the resulting mix with a standard pMHC tetramer protocol or optimized protocol that included PKI and Ab cross-linking (Fig. 8A). The standard protocol failed to recover this functionally efficient T cell, but we were able to recover it with the optimized protocol. We next undertook similar recovery experiments with three more melan A/MART1 clones at a level ∼ 5% CD3^+^ cells. Clones MEL13 and CR29 stain well with standard pMHC tetramer staining and could be recovered very cleanly with the standard protocol (Fig. 8B, middle and bottom panels). In contrast, VB6G4.24 clone, which kills the autologous melanoma cell efficiently (12), could not be recovered by standard pMHC tetramer staining, but it could be recovered with the optimized protocol (Fig. 8B, top panels). Thus, VB6G4.24, which like CR31 is efficient at recognizing the autologous melanoma cell, could not be detected by standard pMHC tetramer staining protocols. Overall, these experiments demonstrate that clones with TCRs revealed by optimized pMHC staining can be functionally relevant and missed by standard staining protocols.

### Clonotypes that require an optimal protocol to stain with tetramer are functionally dominant in patient blood following successful immunotherapy

**FIGURE 9.**
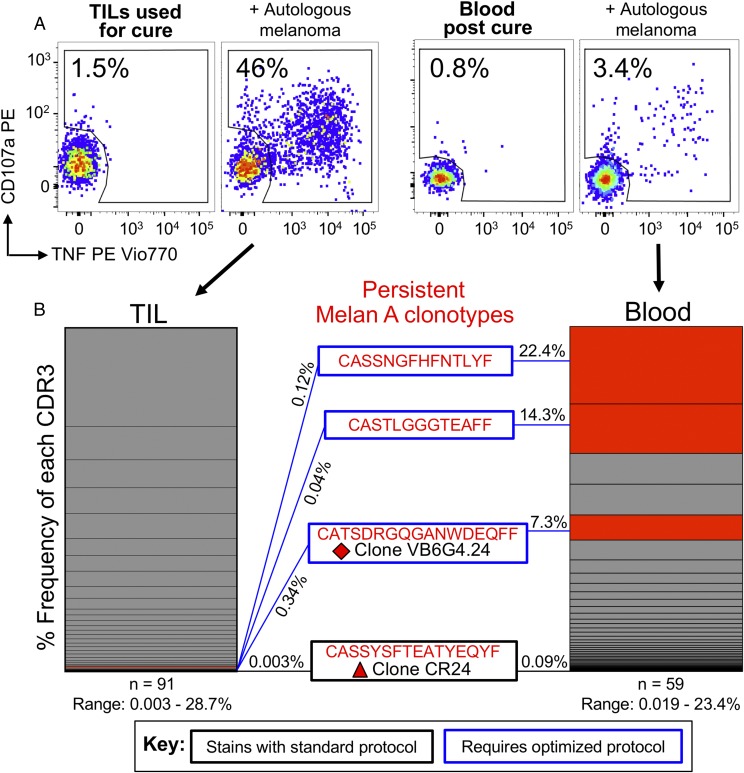
Melanoma-reactive T cells in the blood of a patient cured of cancer are dominated by clonotypes that require an optimal protocol to stain with melan A tetramer. (**A**) The clinical TIL infusion product used to induce a complete lasting remission (>5 y and counting) of metastatic melanoma in HLA A2^+^ Patient MM909.24, as well as PBMCs taken after cure, were stimulated with autologous melanoma for 5 h in the presence of TAPI-0 and Abs for CD107a and TNF. Responding T cells (percentage shown in each gate) were sorted by flow cytometry for high-throughout sequencing of the TRBV. Gating: lymphocytes, single cells, and CD3^+^ ViViD^neg^ cells. (**B**) Unique TCR β-chain clonotypes displayed as vertical slices of the respective TIL or blood charts. CDR3β of persistent melan A–specific T cells are shown in red. The blue boxes indicate TCRs that require an optimal protocol to stain, and the black box indicates a TCR that stains with a standard protocol (Fig. 6). CDR3s of clones VB6G4.24 (red rhombus, needs optimal staining) and CR24 (red triangle, stains with standard protocol) appeared in the melanoma-reactive populations in TILs and blood. The VB6G4.24 clonotype was the fifth most frequent of the clones that responded to tumor in patient blood after treatment.

As described above, melanoma patient MM909.24 underwent a complete lasting remission following TIL therapy (39). The TIL infusion product (cryopreserved and without further in vitro passage) used in the clinic to cure patient MM909.24, as well as blood (PBMCs) taken after complete remission, were stimulated with autologous melanoma, and responding cells (TAPI-0 assay) were sorted for clonotypic analysis by TRBV sequencing. Reactivity toward melanoma was 46% for TILs and 3.4% for blood (Fig. 9). Three CDR3s found in TILs and blood (CASSNGFHFNTLYF, CASTLGGGTEAFF, and CATSDRGQGANWDEQFF) originated from T cells with melan A specificity and had required an optimal tetramer protocol to stain in previous experiments (Fig. 6). The last CDR3 is expressed by clone VB6G4.24, which was characterized in Fig. 6 to be an avid killer of autologous melanoma, yet required the optimal protocol to stain. Impressively, these functional clonotypes dominated the response, amassing 44% (22.4, 14.3, and 7.3%, respectively) of all tumor-reactive CDR3s in the blood of patient MM909.24 after complete remission, which represented a substantial enrichment compared with the infused TILs (0.12, 0.04, and 0.34%, respectively) (Fig. 9). The CDR3 (CASSYSFTEATYEQYF) of melan A clone CR24 (Fig. 6) was also seen, but at a much lower frequency (0.003% in TILs and 0.09% in blood). CR24 exhibits good cytotoxicity toward autologous melanoma and stains with tetramer under standard conditions (Fig. 6). Although not the primary aim of this study, the experiments conducted in Figs. 6 and 9 allowed us to define the specificities (HLA A2, melan A E**L**AGIGILTV) of the two dominant persistent melanoma-reactive clonotypes found in the blood of patient MM909.24 after complete remission. In summary, melanoma-reactive T cells that could only be visualized using optimal tetramer staining (Figs. 6–8) were shown to be fully functional in in vitro (Figs. 6, 7) and ex vivo (Fig. 9) settings. Additionally, TCR clonotypes associated with these T cells were persistent and dominant in vivo and, therefore, were highly likely to be involved in the lasting remission experienced by patient MM909.24.

### Differences in staining efficacy between standard and optimized pMHC tetramer staining protocols are independent of temperature

We perform our tetramer staining on ice, whereas other researchers sometimes use room temperature or 37°C. Therefore, we compared staining of TIL samples, in which there was a sizable tetramer^+^ population, with HLA A2-EAAGIGILTV and HLA A2-E**L**AGIGILTV at 4°C, room temperature (∼22°C), and 37°C. Higher temperatures were observed to increase the number of CD8^+^Tet^+^ cells slightly (Supplemental Fig. 3A). Importantly, the differences in staining efficacy between standard and optimized protocols were independent of temperature (Supplemental Fig. 3A). Staining of PBMC samples at higher temperatures is known to increase nonspecific background (18) (Supplemental Fig. 3B). The signal/noise ratio of preproinsulin_15–24_ dextramer-stained PBMCs from patients with type 1 diabetes was greatly reduced when performed at 37°C; hence, we favored staining on ice.

### Standard pMHC tetramer staining fails to detect functional tumor-specific T cell clonotypes in direct ex vivo PBMC samples

The above experiments with tumor-specific T cells were undertaken with cultured TILs. We next examined T cells specific for a different self-epitope in direct ex vivo PBMCs, a situation in which pMHC tetramer staining is more commonly used. For these studies, we selected IMP2, which is a member of a family of three conserved oncofetal Ags that is known to be very highly expressed between the zygote and embryo stages (52) IMP2 is also known to be expressed by some cancers (53–55), and there are hypotheses that the IMPs, especially IMP3, have an oncogenic role, although this role appears to have stemmed from association with aggressive and advanced cancers rather than any mechanistic insight (56, 57). More recent reports have suggested that IMP2 acts as a tumor promoter by stabilizing oncogenic mRNAs encoding *HMGA1* (58), preventing let-7 target gene silencing (59) and it can be crucial for preserving cancer stem cells in an in vitro model system (60). We have determined that residues 366–376 of IMP2 (NLSALGIFST) are presented on the surface of HLA A2^+^ cells expressing IMP2 and represent a novel HLA A2–restricted epitope (G. Dolton, C. Rius, S.A.E. Galloway, B. Szomolay, V. Bianchi, A. Wall, M. Donia, P. thor Straten, I.M. Svane, and A.K. Sewell, manuscript in preparation).

**FIGURE 10.**
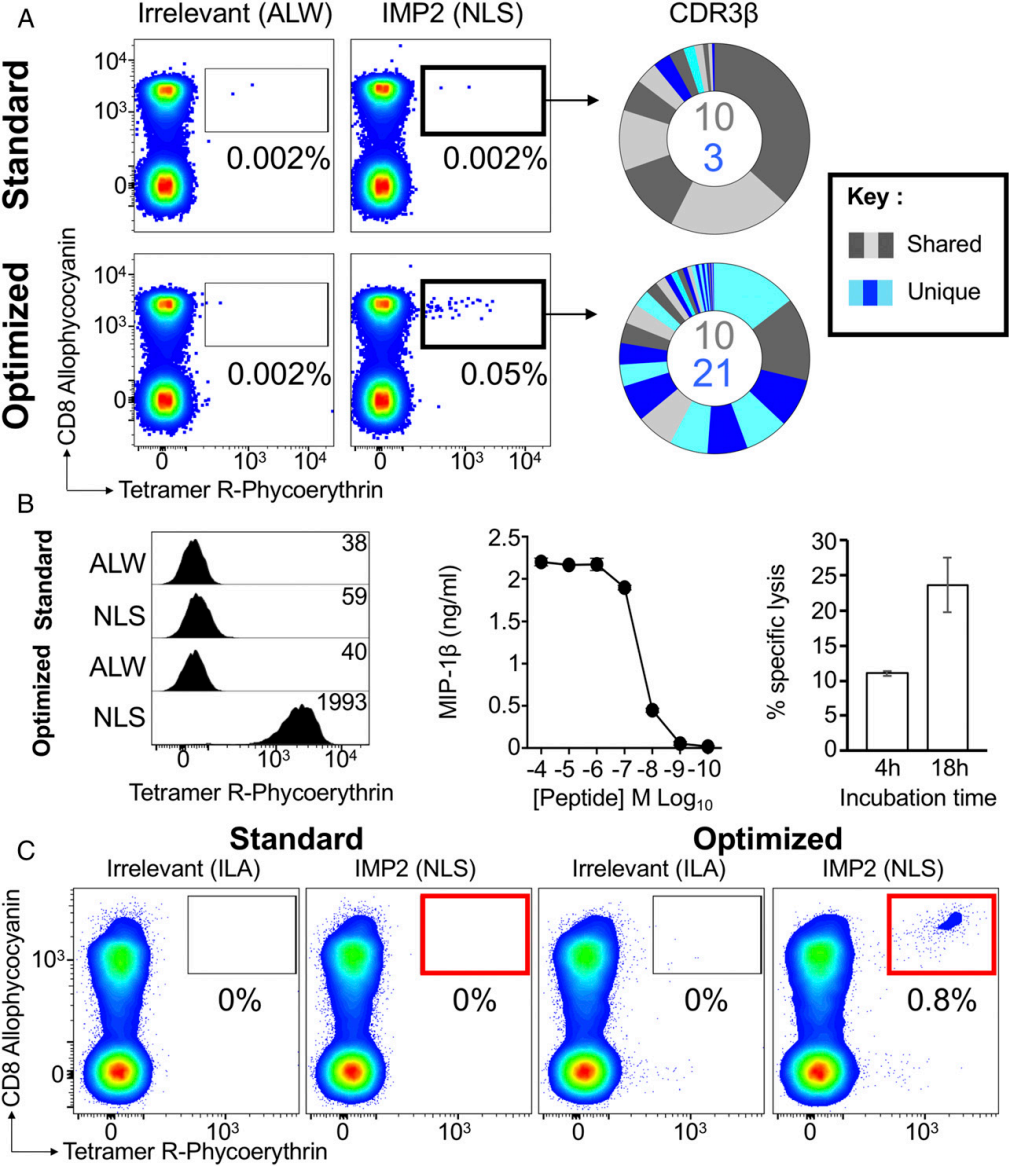
Optimized pMHC multimer staining revealed low-affinity Ag-specific cells. (**A**) PBMCs from HLA A2^+^ Donor 0439 were stained ex vivo by flow cytometry in parallel using HLA A2 IMP2_366–376_ (NLSALGIFSTG) peptide tetramer alone (standard) or in combination with PKI and anti-fluorochrome Ab (optimized). The percentage of tetramer^+^ cells of CD8^+^ T cells is shown for each gate. Irrelevant tetramer made with HLA A2–ALWGPDPAAA preproinsulin-derived peptide (residues 15–24) (66) was used to set the gates for sorting. CD8^+^Tet^+^ cells were sorted for TCR sequencing and CDR3 analysis of β-chains (right panel). Clonotypes are displayed as sort-shared (gray) or sort-unique (blue) sections of a pie, with each section for each sort corresponding to a different CDR3. The number of shared (gray) and unique (blue) CDR3s for the respective sorts are shown in the center of each pie. (**B**) CR0439.NLS CD8^+^ T cell clone grown from Donor 0439’s PBMCs stained weakly for the cognate Ag with the standard protocol and showed improved binding to tetramer with the optimized protocol (left panel). HLA A2–ALWGPDPAAA irrelevant tetramer was used as control. Mean fluorescence intensities are displayed according to the key. The clone responded well to cognate peptide (middle panel), as assessed by MIP-1β release, and exhibited killing of IMP2-expressing melanoma cells (right panel) in a chromium-release assay after 4 and 18 h. (**C**) Recovery of CR0439.NLS clone spiked (aiming for 1% of CD3^+^ cell) into HLA A2^neg^ PBMCs by standard (red box) or optimized (red box) staining protocols with HLA A2–NLSALGIFSTG and irrelevant HLA A2–ILAKFLHWL (hTERT_540–548_) tetramers.

To determine whether our optimized pMHC staining methodology could identify tumor-specific T cells in direct ex vivo PBMCs that were not detected by standard pMHC tetramer staining, we performed ex vivo sorts with the HLA A2–restricted IMP2 sequence NLSALGIFST. Using the strategy outlined in Fig. 1, only 0.002% of cells stained with cognate pMHC tetramer in comparison with 0.05% staining with the optimized protocol (Fig. 10A, left panel). Analysis of TCR β-chain repertoires used by CD8^+^Tet^+^ sorted cells by high-throughput sequencing revealed 13 CDR3s for the standard tetramer–stained cells (69 cells sorted) and 31 CDR3s for the optimized staining (324 cells sorted). Twenty-one unique CDR3β sequences were detected with the optimized protocol (Fig. 10, right panels). Ex vivo sorts were repeated at a different time point and revealed 15 CDR3s sequences for the standard tetramer–stained cells compared with 91 CDR3s for the optimized tetramer staining from 156 and 731 sorted cells, respectively (Supplemental Fig. 4). As before, we grew monoclonal T cell populations from the optimally sorted population from donor 0439. Clone CR0439.NLS (TRAV40/TRAJ53; CDR3α, CLTPSGGSNYKLTLF; TRBV11-3/TRBJ2-5; CDR3β, CASAAYGETQYF) failed to stain with the standard pMHC tetramer protocol. Our optimized protocol increased the mean fluorescence intensity of staining of this clone by >30-fold (Fig. 10B). The CR0439.NLS clone responded well to cognate NLSALGIFSTG peptide and killed melanoma cells (MM909.24) expressing IMP2 (Fig. 10B), as confirmed by intracellular staining with anti-IMP2 Ab (data not shown). This fully functional NLSALGIFSTG-specific T cell clone could not be recovered using standard pMHC tetramer staining when it was spiked into PBMCs (Fig. 10C). In contrast, our optimized protocol recovered this clone with ease (Fig. 10C).

## Discussion

The application of pMHC multimer staining has transformed the study of Ag-specific T cell populations (9), and these reagents have been central to a great number of research studies. Several recent, somewhat alarming, reports have indicated that pMHC staining might fail to detect the majority of functional T cells (12, 18, 19) and have prompted suggestions that T cell immunology has become biased toward the type of cells amenable to detection with multimeric pMHC (24). We stained healthy PBMCs with pMHC tetramers specific for HLA A2–restricted epitopes from influenza, CMV, EBV, and IMP2. Additionally, we examined staining for yellow fever virus–specific cells in a vaccinated donor and tumor-specific T cells in the TILs that were used to induce a complete lasting remission in a patient with stage IV melanoma. Several recent studies have suggested that pMHC multimer staining fails to detect fully functional T cells and, thereby, underestimates the size of Ag-specific T cell populations. This failure is thought to be especially prominent when low-affinity TCRs predominate, such as in MHCII-restricted responses or those directed against self-antigens. Antiviral TCRs are known to bind to their cognate pMHC Ags with relatively high affinity (13, 49), and it has been assumed that standard pMHC tetramer staining is proficient at revealing such cells. We made use of an optimized staining protocol that included use of the PKI dasatinib during staining to prevent “unproductive” TCR downregulation without the capture of pMHC multimer (36) and a fluorochrome-specific Ab to cross-link pMHC multimer and reduce its dissociation from the cell surface during cell washing (12). Parallel staining with optimized and standard protocols recovered similar cell populations from the PBMCs of healthy HLA A2^+^ donors stained with pMHC tetramers of the influenza M1 epitope GILGFVFTL, CMV pp65 epitope NLVPMVATV, and EBV LMP2A epitope CLGGLLTMV. Although both protocols recovered similar T cell populations for these Ags, in each case staining with the optimized protocol had the advantage of being considerably brighter without an adverse signal/noise issue. Although pMHC tetramer staining with the above viral epitopes identified similar CD8^+^Tet^+^ T cell populations, this was not the case with all viral epitopes. Optimized staining with the EBV BMLF1_280–288_ epitope GLCTLVAML identified larger populations of CD8^+^Tet^+^ T cells than could be detected with a standard protocol in five of six donors tested. This situation was further pronounced with yellow fever virus–specific T cells. We also demonstrated that regular pMHC tetramer staining of TIL samples failed to identify fully functional clonotypes that recognized low concentrations of cognate peptide and were efficient at killing the autologous tumor. The functionality of these clones was further confirmed by the enrichment of clonotypes requiring optimized pMHC tetramer in the blood of a “cured” patient. Indeed, just three persistent clonotypes, all of which could not be stained with standard pMHC tetramer staining, accounted for 44% of the entire response to tumor in patient blood following cure, suggesting that these clonotypes may have played a major role in cancer clearance.

We extended this finding by demonstrating that tetramer staining with IMP2-specific reagents failed to recover fully functional clonotypes from fresh PBMCs unless our optimized protocol was used. Our results are in accordance with other studies that have found that functional T cells fail to stain with cognate pMHC multimer (19, 24, 25, 61, 62). Our findings also agree with studies showing that higher-order pMHC multimers, such as pMHC dextramers (11) and dodecamers (18), can recover larger functional T cell populations than recovered by pMHC tetramer staining performed in parallel. The proportion of Ag-specific T cells that is missed by regular pMHC tetramer staining varies widely but can be extremely substantial. Fifty to ninety percent of the T cells that could be recovered by staining TILs and PBMCs with HLA A2–E**L**AGIGILTV dextramers in the presence of PKI and Ab cross-linking could not be detected by standard pMHC tetramer staining (12). Overall, we have found that pMHC tetramer staining can miss a substantial proportion of T cells that can be detected with optimized staining in several systems. These findings appear to be consistent with those of other research groups. Lymphocyte choriomeningitis virus glycoprotein– and oligodendrocyte glycoprotein–specific murine CD4 T cell populations were underestimated by 4- and 8-fold by pMHCII tetramers (19); pMHC dodecamers, which have 12 pMHC molecules per reagent, were able to detect 2–5-fold more Ag-specific human and murine CD4 and CD8 T cells compared with the equivalent tetramers (18). Khan et al. (23) showed that very large populations (5% of total CD8 T cells) in a CMV-seropositive donor responded to the HLA A2–restricted peptide VLEETSVML by intracellular cytokine staining, but these cells could not be recovered with HLA A2–VLEETSVML tetramer, and all 14 VLEETSVML-reactive clones isolated in this study failed to stain with the cognate tetrameric pMHC reagent. Other studies also show that T cell function need not correlate with pMHC multimer staining (19–21) and suggest that these reagents may have routinely underestimated the size of Ag-specific T cell populations over the last 20 y. Importantly, the optimized protocol described in this article was observed to recover many more clonotypes than recovered by standard protocols, including those proven to be functionally relevant. It remains to be seen whether an optimized protocol that includes high-valency pMHC multimers, dasatinib treatment, and pMHC cross-linking with Ab can recover all of the T cells capable of responding to a particular Ag. However, because dasatinib treatment and pMHC cross-linking can be applied to any pMHC multimer stain at a cost < $0.05 per sample, we recommend that this “control” is always used to maximize T cell recovery (or to confirm that it is already maximal). Our results with Donor 0439 and pMHC tetramers of the EBV BMLF1_280–288_ epitope HLA A2–GLCTLVAML were particularly noteworthy, because repeated attempts to stain this EBV-seropositive donor with this tetramer failed >15 y ago. Therefore, we assumed that this donor did not respond to this epitope. Prescreening of HLA A2^+^ PBMCs from laboratory-based healthy donors by ELISPOT indicated that as many as 0.1% of the T cells in PBMCs from Donor 0439 responded to GLCTLVAML peptide. Re-examination with standard pMHC tetramer staining for this study confirmed that these cells stained extremely poorly. Application of our optimized technology, which was unavailable at the time of our initial screening, showed that 0.15% of CD3^+^ cells stained with HLA A2–GLCTLVAML tetramer. A clone grown from this population recognized low concentrations of cognate peptide in the context of HLA A2 and responded to Donor 0439’s autologous EBV-infected LCL. This fully functional T cell clone could not be recovered using standard pMHC tetramer staining when it was spiked into PBMCs, but it was easily distinguishable when we applied our optimized protocol.

It has been suggested that the extensive use of pMHC multimer staining over the last 20 y may have introduced a bias that has continually underestimated the lower-affinity, but functional, components within diverse Ag-specific TCR repertoires (24). Accumulating evidence suggests that T cells with very low–affinity TCRs can make important contributions to immunity in vivo (24, 25, 61–63). Further work will be required to understand how these weak interactions, which could challenge simplistic kinetic proofreading models of TCR triggering (64), are able to precipitate T cell activation.

## Supplementary Material

Data Supplement
