# Supplementary material for: Peptide–MHC Class I Tetramers Can Fail To Detect Relevant Functional T Cell Clonotypes and Underestimate Antigen-Reactive T Cell Populations
Source: J Immunol. 2018 Feb 26;200(7):2263–79. doi: 10.4049/jimmunol.1700242 (PMC5857646; doi:10.4049/jimmunol.1700242)
Supplement: Data Supplement [file JI_1700242.zip › JI_1700242_Supplemental_Figures_1.pdf]

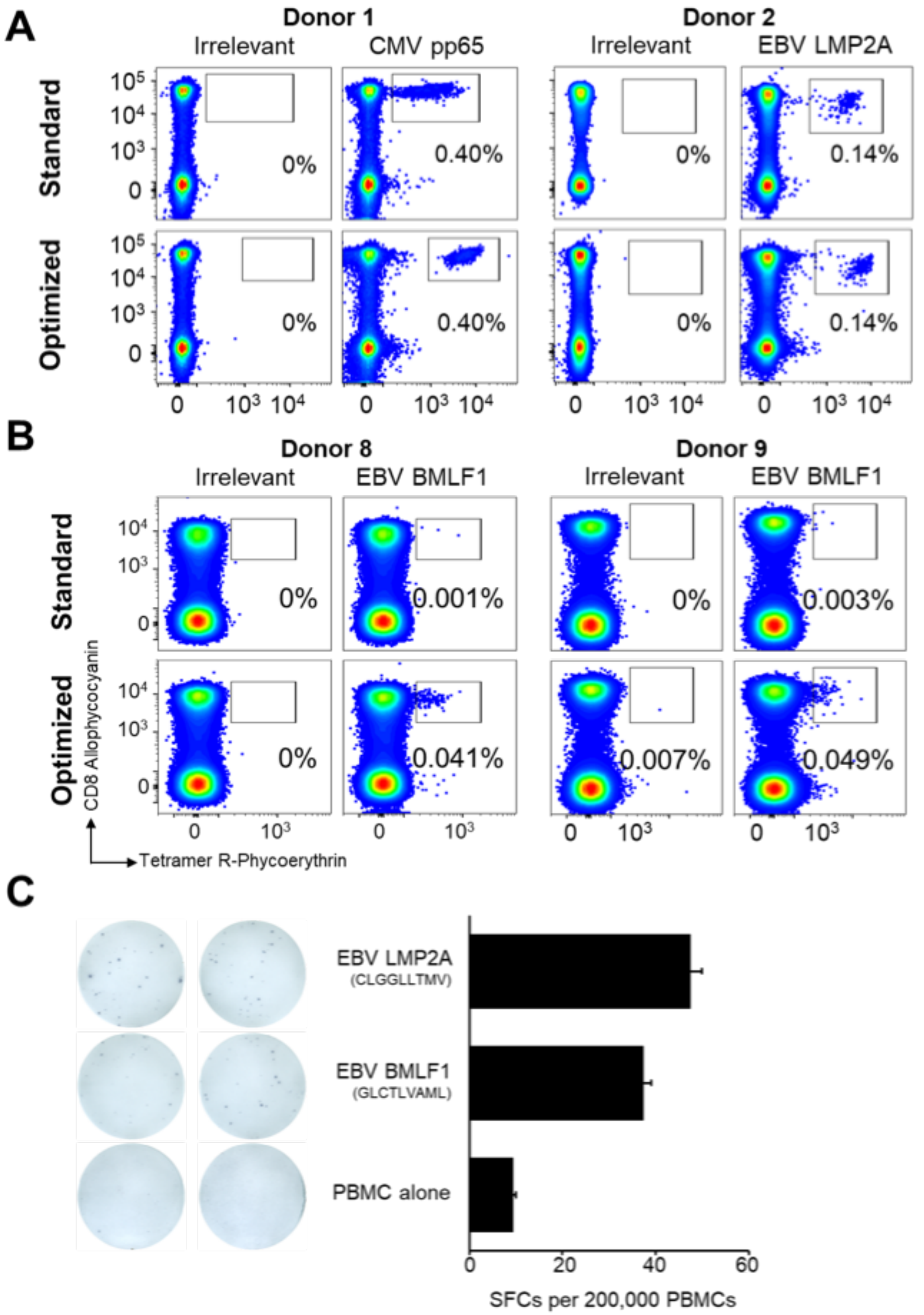

Supplementary Figure 1. *Ex vivo* detection of antigen-specific CD8 T-cells using pMHC multimer staining technology. PBMC from HLA-A2+ healthy donors were stained with tetramers bearing **(A)** the Cytomegalovirus pp65$_{495\text{-}503}$ peptide (NLVPMVATV, left), Epstein-Bar virus (EBV) LMP2A$_{426\text{-}434}$ peptide (CLGGLLTMV, right), or **(B)** HLA-A2 BMLF1$_{280\text{–}288}$ either using tetramer alone (standard) or in combination with PKI and anti-flurorochrome antibody (optimized). Gates were set on lymphocytes and live CD3^{+} CD14^{-} CD19^{-} cells. Irrelevant tetramer made with preproinsulin-derived peptide (PPI$_{15\text{-}24}$, ALWGPDPAAA) or Human Telomerase Reverse Transcriptase (hTERT$_{540\text{-}548}$, ILAKFLHWL) were used to set the gates. Percentage of CD8^{+} tetramer^{+} T-cells is shown for each gate. **(C)** 0439 donor was pre-screened for T-cell activation against virus stimulation. *Ex vivo* PBMC were incubated with EBV-derived CLGGLLTMV and GLCTLVAML peptides (10^{-5} M, in duplicate) overnight. Response was quantified by IFNγ ELISpot. No peptide was used as negative control.

	Donor 5 EBV (GLC)		Donor 0439 EBV (GLC)		Donor 0345 YF (LLW)		TIL Mel (ELA)		Donor 0439 IMP2 (NLS)		Donor 0439 IMP2 (NLS)	
**TRBV1**												0.08
**TRBV2**			0.94								0.84	1.28
**TRBV3-1**				6.71	2.29	4.26			0.43	3.93		2.60
**TRBV3-2**												
**TRBV4-1**				2.41		2.19		1.10	15.91	5.42	15.43	6.65
**TRBV4-2**				0.48				1.00				
**TRBV4-3**												
**TRBV5-1**			14.95	14.87	2.13	0.92			2.59	0.73	2.02	1.67
**TRBV5-2**												
**TRBV5-3**												
**TRBV5-4**			2.30	1.16								0.07
**TRBV5-5**				4.22								0.35
**TRBV5-6**				0.80						0.41		0.57
**TRBV5-7**												
**TRBV5-8**												
**TRBV6-1**				2.83				0.60		0.55		2.64
**TRBV6-2**				0.06								0.35
**TRBV6-3**							5.30	1.10				
**TRBV6-4**												
**TRBV6-5**				4.45	0.71		8.80	1.10				9.43
**TRBV6-6**				5.90								
**TRBV6-7**												
**TRBV6-8**												
**TRBV6-9**												
**TRBV7-1**												
**TRBV7-2**				2.60					37.36	14.42	27.80	0.59
**TRBV7-3**				1.53		0.86						0.52
**TRBV7-4**												
**TRBV7-5**												
**TRBV7-6**				1.42				1.10		0.70		0.85
**TRBV7-7**												
**TRBV7-8**				3.29						0.17		2.42
**TRBV7-9**			35.53	5.56					5.46	16.61	3.07	8.26
**TRBV8-1**												
**TRBV8-2**												
**TRBV9**			0.65	5.49							2.61	0.40
**TRBV10-1**											6.93	7.43
**TRBV10-2**												0.99
**TRBV10-3**				2.02								0.48
**TRBV11-1**												
**TRBV11-2**			5.62	3.34								6.15
**TRBV11-3**												0.11
**TRBV12-1**												
**TRBV12-2**												
**TRBV12-3**				5.05								1.09
**TRBV12-4**			6.46	2.00			5.30	3.10				2.94
**TRBV12-5**										8.28		1.08
**TRBV13**									1.43	0.17	0.37	1.24
**TRBV14**				0.11								
**TRBV15**				0.75	38.83	44.91		0.70		0.17		0.09
**TRBV16**				0.24								
**TRBV17**												
**TRBV18**				0.05						7.37	0.34	18.60
**TRBV19**			13.73	0.40						0.55		0.34
**TRBV20-1**			19.81	8.05	52.17	35.36		2.70	24.70	11.31	10.68	1.40
**TRBV21-1**												
**TRBV22**												
**TRBV23-1**												
**TRBV24-1**				0.54				2.80		0.79	18.98	7.15
**TRBV25-1**				0.78								
**TRBV26**												
**TRBV27**	0.04	0.08		4.46	3.87	6.38	68.40	64.20	12.12	13.84	8.70	4.91
**TRBV28**				2.49		2.07		9.70				0.38
**TRBV29-1**	99.96	99.92		5.91		2.65		2.40		14.57	2.24	6.85
**TRBV30**							12.30	5.00				

Key:
- Standard Protocol
- Optimised Protocol

Supplementary Figure 2. Cumulative %TRBV gene usage for standard (Yellow) and optimized (blue) staining protocols, with predominant gene usages indicated by darker colors in the heatmap. All human genes listed on the Y-axis for completeness.

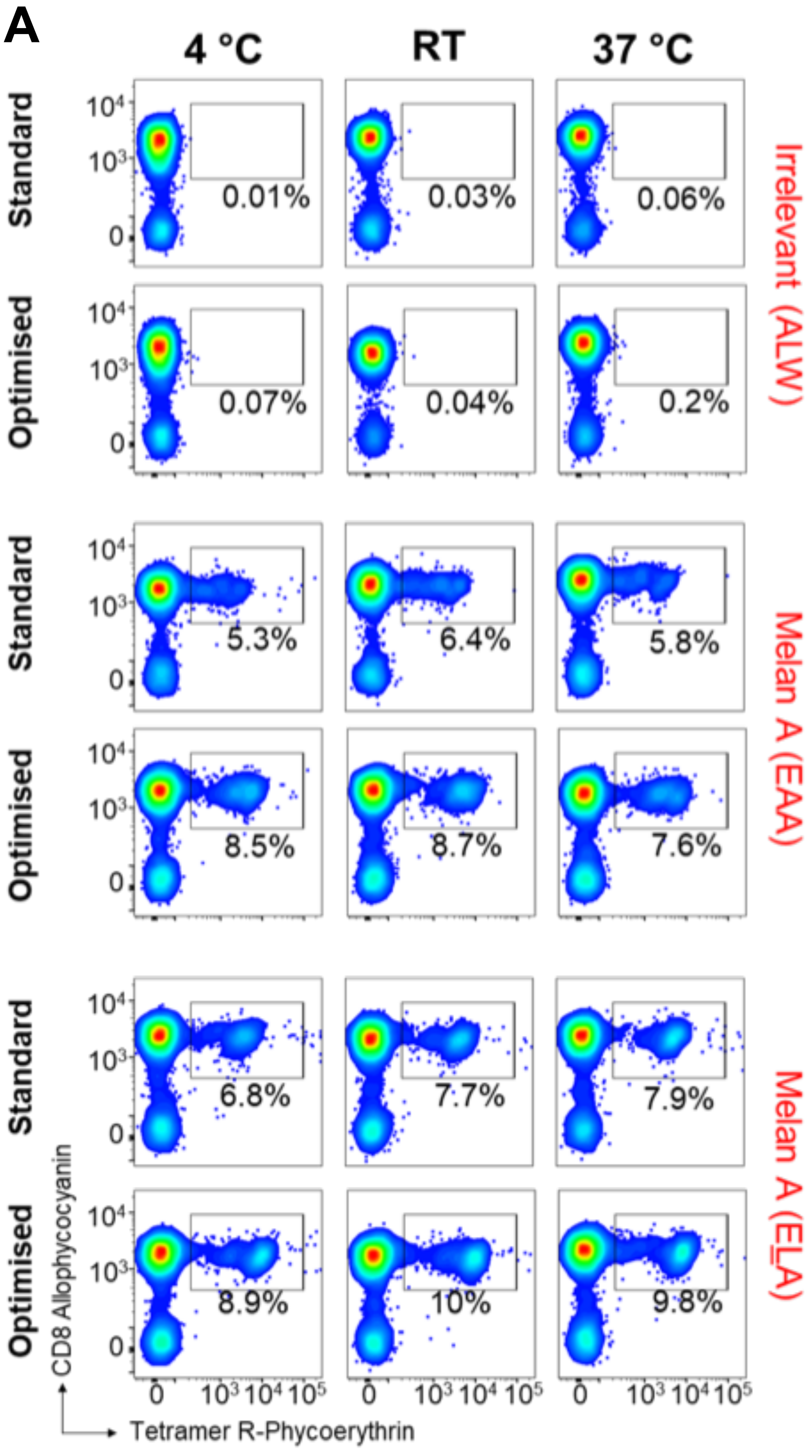

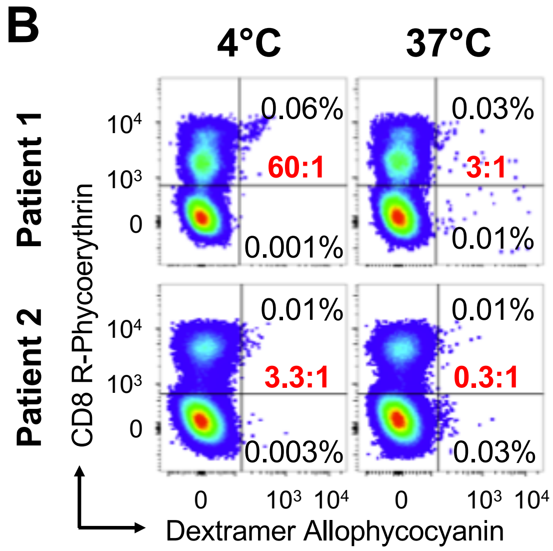

Supplementary Figure 3. (A) Melan A specific TILs were stained using HLA A2-EAAGILGILTV and HLA A2-ELAGILGILTV standard or optimized tetramer staining at 4°C, 37°C or room temperature (~22 °C). The percentage of tetramer⁺ is displayed. Irrelevant HLA A2-ALWGPDPAAA (preproinsulin residues 15-24) was used to set the gates. **(B)** Peripheral blood monocular cells from HLA A2⁺ donors with type I diabetes were thawed and stained with HLA A2-ALWGPDPAAA (preproinsulin residues 15-24) allophycocyanin conjugated dextramer. Staining was performed on ice (4°C) or at 37°C. The percentage of viable CD3 cells is shown for the CD8⁺ dextramer⁺ (upper right) and CD8^{neg} control (lower left) gate, with the signal to noise ratio in red.

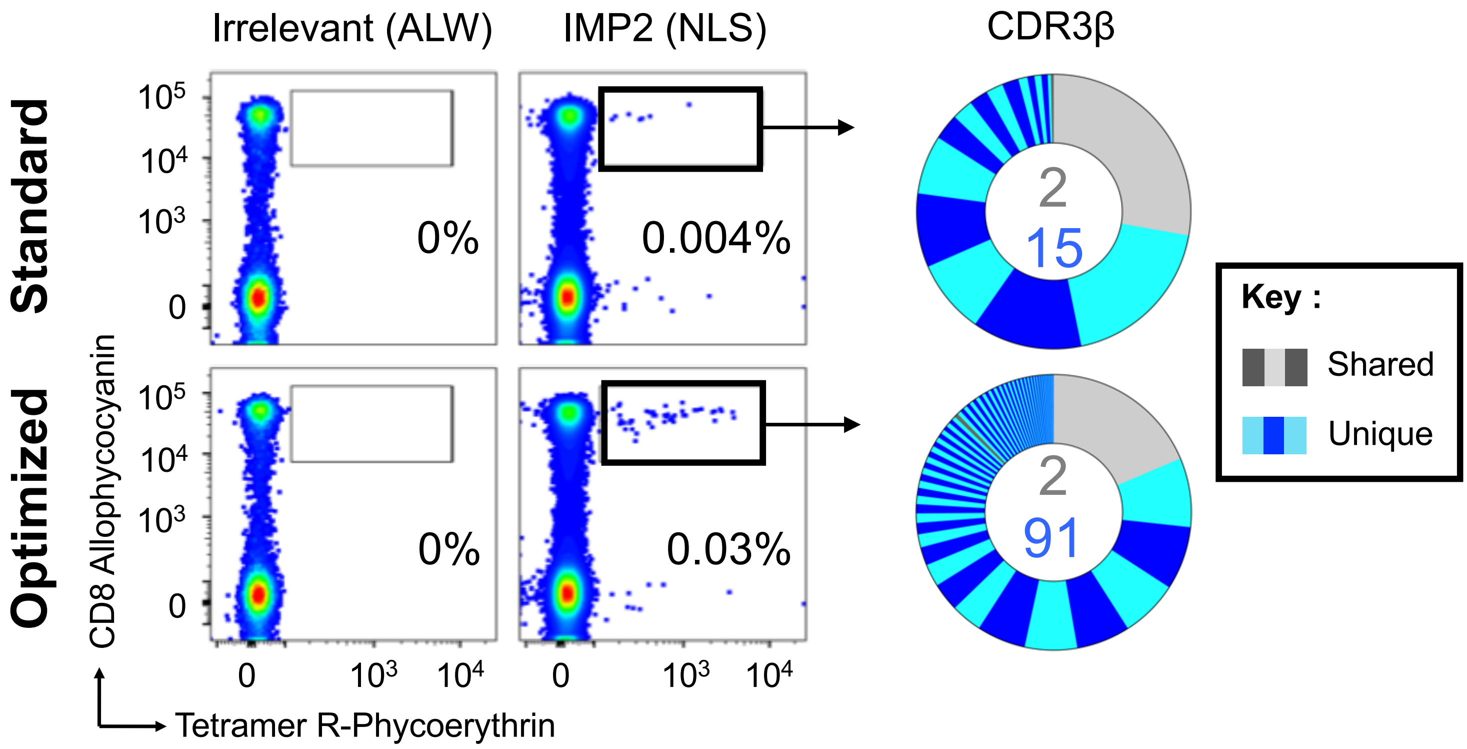

Supplementary Figure 4. Optimized pMHC multimer staining results are reproducible. (A) PBMC from donor 0439 were *ex vivo* stained in parallel using either IMP2 standard or optimized tetramer protocols Percentage tetramer$^+$ cells of CD8$^+$ T-cells is shown for each gate. Irrelevant tetramer made with PPI was used to set the gates for sorting. CD8$^+$ Tetramer$^+$ cells were sorted for TCR sequencing and CDR3 analysis of β chains (right). Clonotypes are displayed as sort-shared (grey) or sort-unique (blue) sections of a donut pie, with each section for each sort corresponding to a different CDR3. The number of shared (grey) and unique (blue) CDR3s for the respective sorts are shown in the center of each pie.
